# Gut microbial metabolite butyrate suppresses hepatocellular carcinoma growth via CXCL11-dependent enhancement of natural killer cell infiltration

**DOI:** 10.1080/19490976.2025.2519706

**Published:** 2025-06-27

**Authors:** Menghan Zhang, Xuefeng Huang, Yanlong Zhang, Minghang Yu, Xiaoxue Yuan, Yifan Xu, Lei Ma, Xi Wang, Huichun Xing

**Affiliations:** a Center of Liver Diseases Division 3, Beijing Ditan Hospital, Capital Medical University, Beijing, China; b National Key Laboratory of Intelligent Tracking and Forecasting for Infectious Diseases, Beijing, China; c Beijing Institute of Infectious Diseases, Beijing, China; d National Center for Infectious Diseases, Beijing Ditan Hospital, Capital Medical University, Beijing, China; e Beijing Key Laboratory of Viral Infectious Diseases, Beijing Ditan Hospital, Capital Medical University, Beijing, China; f Department of Oncology, Capital Medical University, Beijing, China; g Shanxi Bethune Hospital, Shanxi Academy of Medical Sciences, Tongji Shanxi Hospital, Third Hospital of Shanxi Medical University, Taiyuan, China; h Peking University Ditan Teaching Hospital, Beijing, China

**Keywords:** Gut microbiota, butyrate, epigenetic, natural killer cells, hepatocellular carcinoma

## Abstract

Gut microbiota-derived butyrate plays a vital role in attenuating hepatocellular carcinoma (HCC) in murine models. However, the precise molecular mechanisms by which butyrate exerts its effects are largely undefined. Plasma short-chain fatty acids (SCFAs) were quantitatively measured by using gas chromatography-mass spectrometry (GC-MS) to access their association with HCC prognosis. Tumor-infiltrating immune cells were characterized by flow cytometry. The interactions between butyrate and natural killer (NK) cells were studied using in vitro assays, including migration, cytotoxic degranulation, and co-culture experiments. In vivo validation was conducted through neutralization experiments. The molecular pathways regulated by butyrate were further investigated by employing RNA sequencing (RNA-seq), chromatin immunoprecipitation sequencing (ChIP-seq), and Assay for Transposase-Accessible Chromatin using sequencing (ATAC-seq). A positive correlation was observed between elevated plasma butyrate levels and improved prognosis in HCC patients. Notably, butyrate inhibited tumor progression by enhancing NK cell infiltration into tumor tissues. Mechanistically, butyrate stimulated cytokine secretion, notably significantly enhancing the production of the chemokine CXCL11, thereby facilitating NK cell infiltration. Gene Set Enrichment Analysis (GSEA) of hepatic tumor cell lines revealed that the chemokine signaling and NK cell-mediated cytotoxicity pathways were upregulated following butyrate stimulation. Furthermore, transcriptomic and epigenomic analyses showed that exposure to butyrate induced de novo chromatin accessibility and enhancer remodeling, regulated by STAT family transcription factors. Our study demonstrated that butyrate was able to enhance the expression of CXCL11. This is likely attributed to chromatin remodeling, and then promoting NK cell infiltration and exerting effective anti-tumor effects on HCC.

## GRAPHICAL ABSTRACT

**Figure:**
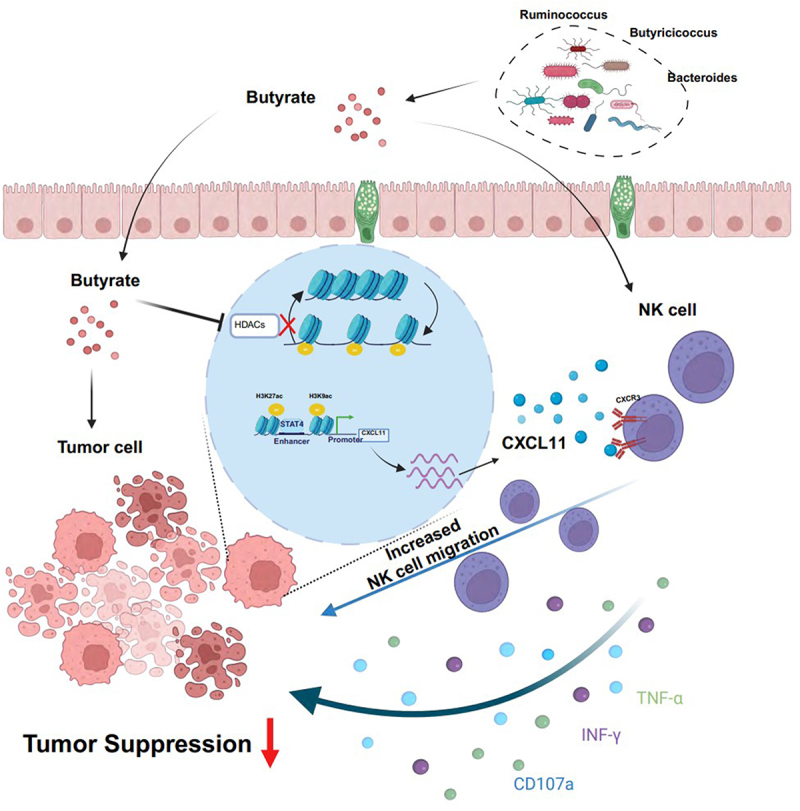

## Introduction

Hepatocellular carcinoma (HCC) represents the sixth most common malignancy and ranks third in terms of cancer-related mortality. Despite recent advancements, the global incidence of HCC has been on the rise, and treatment options are still limited.^1^

The pathogenesis of liver cancer involves various hepatocellular components and extrahepatic factors.^2^ HCC is associated with fibrosis, cirrhosis, chronic hepatitis, and stromal activation, all of which contribute to an inflammatory environment that triggers antitumor immune responses.^3^ Within the tumor immune microenvironment (TIME), immune cells undergo both functional and structural changes, which significantly impact HCC progression, metastasis, and prognosis. Hence, unraveling the diverse roles that infiltrating immune cells play in the TIME is crucial to the effective management of HCC. Moreover, the gut microbiota is emerging as a significant extrahepatic factor in the development and progression of HCC. Throughout this process, the gut microbiota and their metabolites not only shape the pathology of the liver but also intricately regulate both hepatic and systemic immune responses, contributing to the complexity of HCC.^4^ A recent study showed that the gut microbiome worked as a vital regulator of antitumor immunity in melanoma patients.^5^ However, the intricate mechanisms underlying the impact of gut microbiota and their metabolites on liver immunity and tumor development remain elusive.

Short-chain fatty acids (SCFAs), defined as fatty acids containing no more than six carbon atoms, mostly acetate, propionate, and butyrate, are primarily produced in the intestine through bacterial fermentation of indigestible carbohydrates,^6^ which are important for mitigating inflammation and maintaining intestinal homeostasis.^7^ Recent findings suggest that microbiota-produced butyrate can modulate the immune response system, thereby contributing to various health-promoting functions.^8^ Propionate has been demonstrated to alleviate gut inflammation by directly influencing the differentiation of colonic Treg cells.^9^ Acetate has the potential to decrease infiltration of IL-17A-producing ILC3 cells, and anti-cancer therapies in combination with immune checkpoint inhibitors could enhance the efficacy of clinical treatments for HCC.^10^ Previous evidence indicated that SCFAs could impede hepatic tumor growth by regulating gene expression pivotal for tumor cell proliferation and apoptosis.^11^ These findings prompted us to investigate the role of butyrate in shaping the TIME within the context of HCC.

Besides, microbial-derived SCFAs were found to exert anticancer effect in colon cancer,^12^ regulate insulin,^13^ and modulate the immune system^14^ without altering the DNA sequence. Given this, epigenetic changes may play a crucial role in mediating butyrate-mediated transcriptome alterations in HCC.^15^ Epigenetic processes operate at multiple levels of nuclear organization. For instance, posttranslational modifications of histone proteins, such as acetylation, methylation, ubiquitination, or phosphorylation, predominantly take place on the unstructured tails of histone core proteins within nucleosomes.^16^ However, studies on the involvement of histone modifications and distant cis-regulatory elements in butyrate-mediated transcriptional regulation remain limited.

In this study, we investigated the mechanism by which SCFAs, specifically butyrate, exerts an anti-tumor effect through modulating TIME in the liver. The results demonstrated that butyrate could enhance NK cells accumulation through a CXCL11/CXCR3/STAT4 dependent mechanism, This in turn, promoted antitumor immunity in the liver. These findings suggest that the immunomodulatory role of butyrate might exert a pivotal influence on liver cancer.

### Statistical analysis

Data were analyzed by utilizing R and GraphPad Prism 8. Student’s t-test was employed for comparison of normally distributed variables, Mann – Whitney U-test was employed for analysis of non-normally distributed data. For multigroup comparisons, one-way ANOVA and Kruskal – Wallis tests were conducted. Survival analysis was performed by using Kaplan – Meier method with log-rank test, with significance set at a *p* < 0.05 (two-tailed).

## Results

### Characteristics of gut microbiota and SCFAs levels in the HCC patients

**Figure 1.**
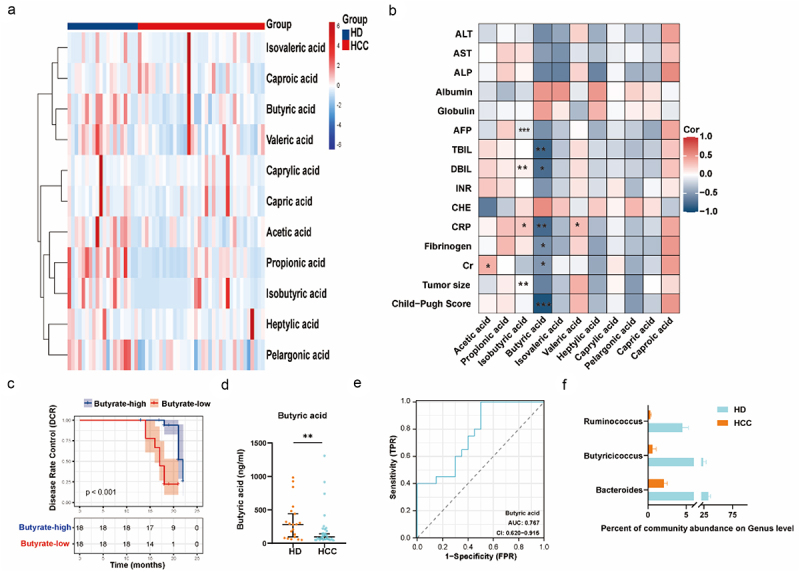
Gut microbiota-derived butyrate is associated with the prognosis of HCC patients. (a) Heatmap illustrating alterations in human plasma short-chain fatty acids (SCFAs) in healthy donors (HD, *N* = 20) and hepatocellular carcinoma patients (HCC, *N* = 36). (b) Spearman correlation analysis showing the association between the concentration of plasma SCFAs and clinical characteristics in HCC patients. *p < 0.05, **p < 0.01, and ***p < 0.001, by Spearman test. (c) Progression-free survival in butyrate-high and butyrate-low groups. *p*<0.001, by log-rank test. (d) GC-MS analysis revealing differences in plasma butyrate level between HD (*N* = 20) and HCC (*N* = 36) groups. (e) Receiver operating characteristic (ROC) analysis distinguishing HCC patients and HD in terms of plasma butyrate. (f) Percentage of community abundance at the phylum and genus level of gut microbiota in the two studied groups. **p < 0.01, by Mann-Whitney test.

Bacterial DNA from fecal samples of hepatocellular carcinoma (HCC) patients (*n* = 36) and healthy donors (HD, *n* = 20) were extracted and subjected to 16S ribosomal RNA gene sequencing for taxonomic profiling. Demographics and clinical features of HCC patients and healthy subjects are detailed in Supplemental Table S1. The α-diversity (Chao1, Simpson, and Shannon indices) and β-diversity (non-metric multidimensional scaling, NMDS) analyses revealed a decreased taxonomic diversity in the microbiota of HCC patients compared to HD. (Figure S1A, and Figure S1B). To explore differences in fecal microbial composition between HCC patients and HD, linear discriminant analysis effect size (LEfSe) was performed (Figure S1C). Notably, at the genus level, *Bacteroides* and *Prevotella_9* were decreased, while *Escherichia-Shigella*, *Klebsiella*, and other pathogenic species such as *Erysipelatoclostridiaceae*, *Veillonellaceae*, and *Streptococcaceae* were significantly increased in HCC patients (Figure S1D). These alterations suggest a potential link between gut microbiota and HCC pathogenesis.^4^ However, more thorough examination is necessary to delve into the underlying mechanisms. Short-chain fatty acids (SCFAs), synthesized in the colon and metabolized by enzymes derived from intestinal bacteria, play a crucial part in maintaining a healthy gut microbiota, innate immunity, and an equilibrium in lipid and carbohydrate metabolism.^17^ In this study, we conducted gas chromatography-mass spectrometry (GC-MS) on plasma samples obtained from HCC patients or healthy donors (Figure 1(a)), and an orthogonal partial least squares-discriminant (OPLS) analysis revealed the SCFA profile of HCC patients was distinctively different from that of HD, as illustrated in Figure S1E. Additionally, Spearman’s correlation coefficient between differential SCFA metabolites and clinical indices of HCC patients was analyzed and presented as a heat map. It is interesting that plasma butyrate levels exhibited the most significant negative correlation with clinical indices of HCC (Figure 1(b)). In addition, butyrate was an independent factor influencing HCC outcomes, rather than being confounded by liver function-related factors (Supplemental Table S2 and S3). Furthermore, among patients with comparable tumor features and treatment modalities, those with higher-level plasma butyrate experienced a better progression-free survival than their low-level counterparts (Figure 1(c)). In addition to butyric acid, other plasma SCFA-such as acetic acid, propionic acid, and pelargonic acid-were all significantly reduced in HCC patients (Figure 1(d) and Figure S1F). The ROC curve analysis suggested that plasma butyrate levels may be strongly associated with HCC incidence, with an area under the ROC curve (AUC) of 0.767 (Figure 1(e)). Additionally, certain butyrate-producing bacteria such as *Ruminococcus*, *Butyricicoccus*, and *Bacteroides*, were significantly decreased in HCC patients (Figure 1(f)).

### Butyrate treatment inhibits tumor growth and enhances NK cell infiltration

**Figure 2.**
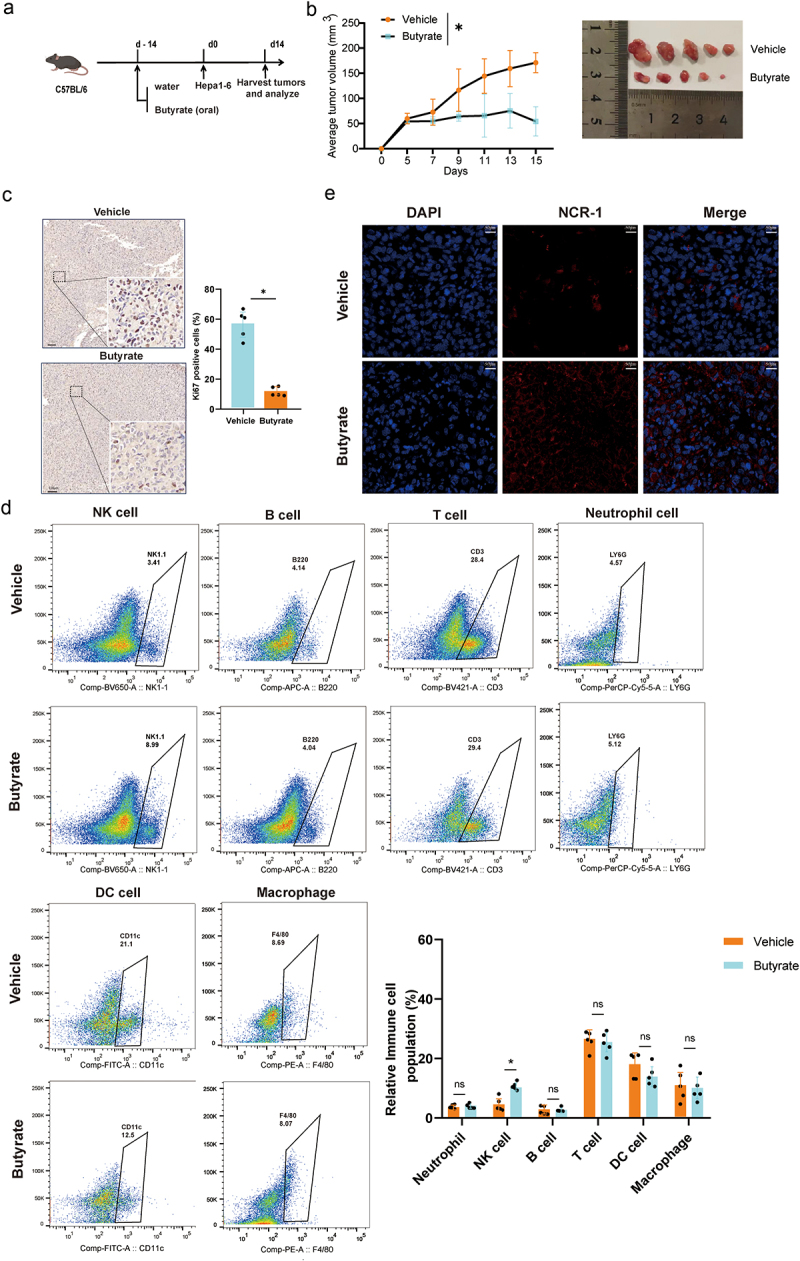
Butyrate exposure significantly attenuates tumor growth and reshapes the tumor microenvironment. (a) Schematic overview of butyrate treatment in Hepa1–6-bearing mice. *N* = 5 mice for each group. (b) Tumor volume was monitored, and representative images shown on the right. *N* = 5 mice per group. (c) Representative images of Ki67-positive cells of tumor sections in the control and butyrate-treated group (scale bar, 100 μM). Quantification of Ki67^+^ in the tumor (right). *N* = 5 mice per group. (d) C57BL/6 mice were pretreated with or without butyrate feeding as described in methods. Tumor-infiltrating immune cells were measured by flow cytometry. *N* = 5 mice per group. Data are expressed as mean ± SEM. *p < 0.05, **p < 0.01, by two-tailed Student’s t-test. (e) Immunofluorescence staining of tumor tissues with anti- NCR1.

To assess the impact of butyrate on liver tumor growth and explore its potential mechanism, C57BL/6 mice were given either plain drinking water or water supplemented with butyrate, followed by subcutaneous inoculation of hepa1–6 hepatic cancer cells. After a 14-day period, mice tumor tissues were collected for flow cytometric analysis. In addition, we also harvested the peripheral blood and feces from mice for the determination of butyrate levels and found that the butyrate level in the butyrate-fed mice was significantly increased in peripheral blood and feces compared to the control group, with the difference being statistically significant (Figure S2). Notably, oral butyrate reduced the tumor volume in C57BL/6 mice treated with butyrate compared to water-only controls (Figure 2(a,b)). Ki67^+^ tumor cells were diminished in butyrate-treated mice (Figure 2(c)). Further, flow cytometry demonstrated a significant increase in NK1.1^+^ NK cells within tumor tissues following butyrate treatment, with minimal impact found on the populations of F4/80+ macrophages, CD3+ T lymphocytes, B220+ B lymphocytes, and Ly6G+ neutrophils (Figure 2(d)). Ultimately, NCR-1 immunofluorescence staining of tumor tissues from mice indicated that butyrate triggered an infiltration of NK cells into the tumor tissues (Figure 2(e)). Collectively, these findings suggest that butyrate treatment impedes tumor development and enhances NK cell recruitment in the TIME.

### Butyrate-induced tumor suppression relies on NK cells

**Figure 3.**
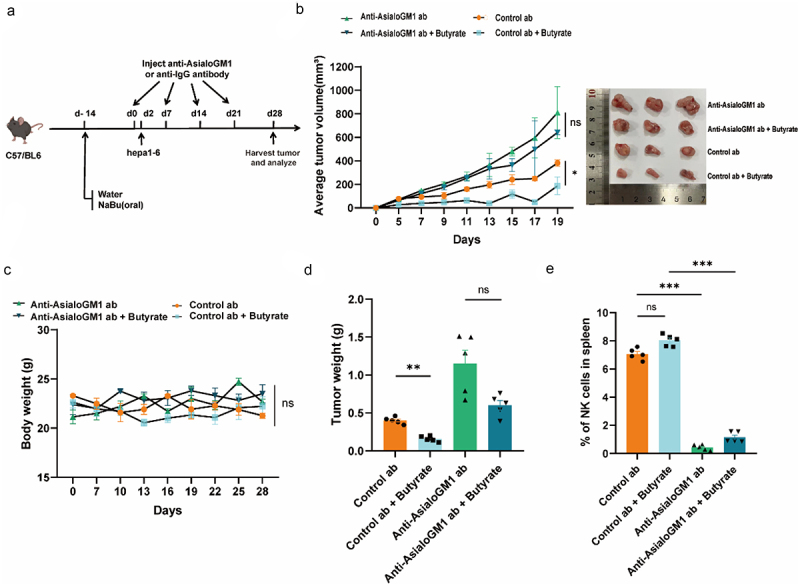
NK cells are essential for butyrate-mediated suppression of hepatic tumor growth in a mouse model. (a) Schematic illustration of the experimental design and timeline for NK cell depletion assays in mouse models. (b) Monitoring of average tumor sizes under different conditions since tumor inoculation. *N* = 5 mice for each group. Representative images are displayed on the right. (c) Weight curves for mice. *N* = 5 mice for each group. (d) Recording of average tumor weight. *N* = 5 mice for each group. (e) Flow cytometrical measurement of the relative percentage (%) of NK cells in spleens from mice. *N* = 5 mice for each group. Data are presented as mean ± SEM. *p < 0.05, **p < 0.01, ***p < 0.001, two-tailed Student’s test.

Considering the increased infiltration of NK cells and the relatively low proportion of apoptotic tumor cells in tumor tissues treated with butyrate (Figure 2(d,e), Figure S3), we proposed that the *in vivo* suppression of Hepa1–6 tumors by butyrate depends on the presence of NK cells. To illustrate this, we treated mice with an NK cell-depleting antibody, anti-ASGM1, and flow cytometry analysis confirmed a highly efficient depletion of NK cells (CD3^−^/NK1.1^+^/NKP46^+^) in the anti-ASGM1 group compared to the isotype IgG control group (Figure 3(a–e)). Interestingly, the tumor-suppressing effect of butyrate was significantly reduced in NK cell-depleted mice compared to those with normal NK cell levels. Nonetheless, this phenomenon was not observed in the tumor-bearing mice treated with the vehicle. (Figure 3(b–d)). Furthermore, no significant differences in body weight were observed among the mice treated with different antibodies (Figure 3(c)).

To further confirm the direct effect of butyrate on NK cells, single-cell suspensions from mice spleen were prepared, isolated, and stimulated with Phorbol 12-Myristate 13-Acetate (PMA) and ionomycin (INSO) *in vitro*, with or without butyrate treatment. The results showed that butyrate treatment up-regulated the expression of CD107a and IFN-γ in NK cells (Figure S4A). Additionally, our research demonstrated an up-regulated expression of natural killer group 2D (NKG2D) on hepatic tumor cells after butyrate stimulation (Figure S4B). The lactate dehydrogenase (LDH) assay, which measures NK cell-mediated cytotoxicity, showed that butyrate enhanced the cytotoxic effect of NK cells on cancer cells (Figure S4C). Moreover, we also found that butyrate stimulated the release of CD107A and IFNγ from NK cells and significantly enhanced their proliferation (Figure S4D and E). These findings suggest that the tumor-suppressive effect of butyrate is largely dependent on NK cells.

### Butyrate induces NK cell recruitment by upregulating CXCL11 in tumor cells

**Figure 4.**
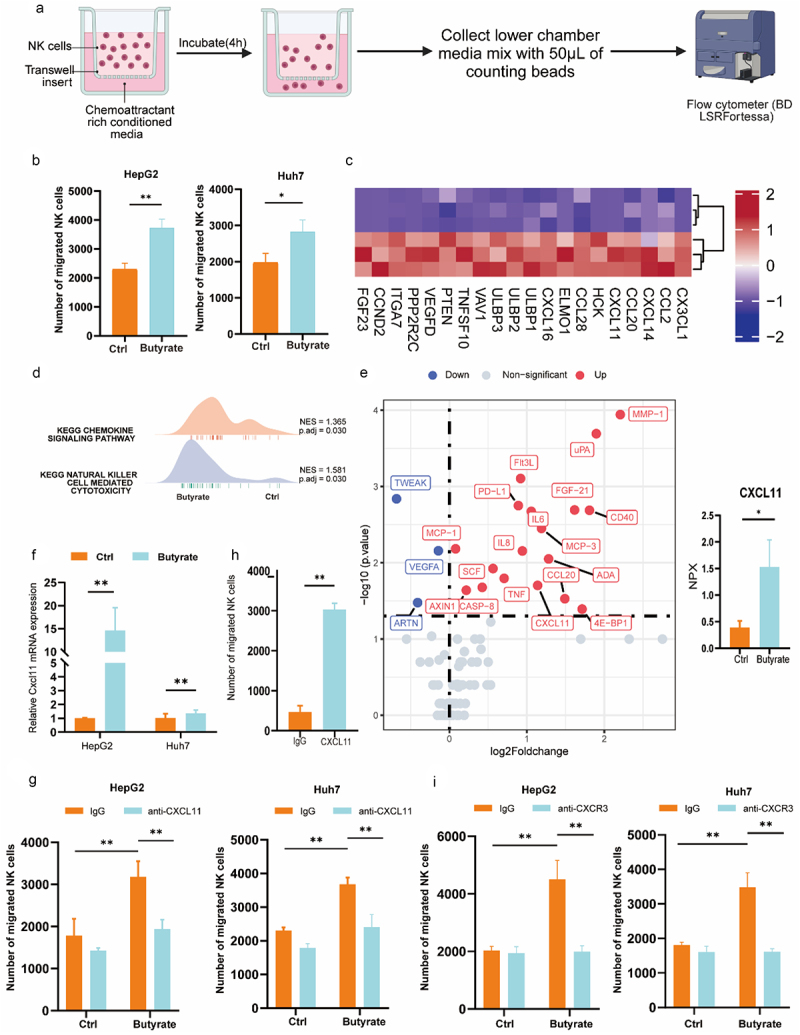
Butyrate-mediated up-regulation of CXCL11 is crucial for NK cell migration. (a) Schematic illustration of NK cell migration assays. (b) Transwell assay showing the NK cells migrating towards conditioned media from HepG2 and Huh7 cells pre-treated with butyrate (1 mm) and PBS for 48 h. (c) RNA-seq analysis of differentially-expressed genes in butyrate-treated hepatic tumor cells (HepG2) for 48 h, with a heatmap illustrating significantly upregulated genes. (d) Gene set enrichment analysis (GSEA) based on RNA-seq profiles, showing a positive association between butyrate treatment and the chemokines signaling pathway and the natural killer cells-mediated cytotoxicity pathway. (e) Analysis of conditioned media from HepG2 cells treated with butyrate or PBS using a 92 inflammation panels, with quantitative assessment of CXCL11 expression displayed using normalization protein expression (NPX) data. (f) qRT-PCR measurement of relative CXCL11 mRNA expression in HepG2 and Huh7 cells treated with butyrate (1 mm) for 48 h compared to PBS-treated cells, with ACTINB as the internal reference. (g) ELISA measurement of CXCL11 protein levels in supernatants from HepG2 and Huh7 cells treated with butyrate (1 mm) or PBS for 48 h. (h) Average number of NK cells that migrated toward conditioned media with or without recombinant human CXCL11 protein. (i) Average number of NK cells that migrated toward conditioned media collected from HepG2 and Huh7 cells that pretreated with butyrate, with or without CXCL11-neutralizing antibodies. Data are given as mean ± SEM based on three independent experiments. *p < 0.05; **p < 0.01; ***p < 0.001, two-tailed Student’s test.

Based on these observations and the well-established role of chemokines in orchestrating immune responses against cancer cells, we investigated whether butyrate could modulate chemokine expression to influence NK cell migration. To test this hypothesis, a transwell assay was performed to assess the effect of conditioned media from butyrate-treated hepatic tumor cells on NK cell migration (Figure 4(a)). Since butyrate has the potential to induce apoptosis in cancer cells,^18^ we tested various concentrations of butyrate and observed that exposing hepatoma cell lines to 1 mm butyrate for 48 hours had minimal effects on proliferation and survival and did not induce apoptosis (Figure S5A – C). This concentration range allows for a more thorough examination of how butyrate influences the interaction between immune cells and tumor cells. In these experiments, we initially treated two hepatic tumor cell lines, HepG2 and Huh7, with either butyrate or PBS (serving as control). Subsequently, conditioned media from the treated cells were collected to measure NK cell migratory activity. The results revealed that conditioned media from hepatic tumor cells treated with butyrate significantly enhanced NK cell migration compared to media from PBS-treated cells (Figure 4(b)).

To further investigate the mechanisms behind butyrate-induced NK chemotaxis, HepG2 cells-with or without butyrate treatment-underwent RNA sequencing (RNA-seq). Following butyrate treatment, numerous genes were upregulated, including several genes associated with chemokines (CX3CL1, CCL2, CXCL11, CXCL8, CCL20, CXCL16, CCL26), NK cytotoxicity (ULBP1, ULBP2, ULBP3, *etc*.), and cell proliferation (PTEN, CCND2, ITGA7, etc.) (Figure 4(c)). This suggests that butyrate may enhance the immune response against cancer cells not only by upregulating chemokine expression to promote NK cell migration and by enhancing NK cell cytotoxicity. Gene set enrichment analysis (GSEA) revealed significant enrichment in the “chemokine signaling pathway” and “natural killer cell cytotoxicity” pathways (Figure 4(d)), suggesting a link between the phenotypic changes observed after butyrate treatment and these pathways.

Based on these findings and the well-established roles of chemokines in the regulation of immune responses against cancer cells, we investigated whether butyrate could modulate chemokine expression to influence NK cell migration. To test this hypothesis and identify butyrate-regulated chemokines, we treated the hepatic tumor cell line HepG2 with butyrate or PBS for 48 hours. Subsequently, we used the Olink Target 96 Inflammation panel with proximity extension assays (PEA) to measure levels of 92 protein markers. Our analysis identified significant changes in 21 out of 92 proteins in the butyrate-treated group, including tumor necrosis factor (TNF), interleukins, and chemokines (Figure 4(e)). Notably, CXCL11, MCP-1, and CCL20 were up-regulated in butyrate-treated cells and exhibited distinct chemotactic activities.

Given previous reports linking high MCP-1 and CCL20 levels to unfavorable prognosis in HCC patients,^19,20^ along with the known role of CXCL chemo-attractants in immune cell trafficking,^18^ and the paradoxical role of CXCL11 in HCC pathogenesis, we focused on CXCL11 regulation by butyrate for the remainder of the study. qRT-PCR confirmed that treatment of two distinct hepatic tumor cell lines with butyrate led to up-regulation of CXCL11 (Figure 4(f)). To assess whether CXCL11 protein levels correlated with mRNA levels, we performed enzyme-linked immunosorbent assays (ELISAs) on conditioned media from HepG2 and Huh7 cells treated with butyrate. As anticipated, the level of CXCL11 protein was also significantly increased in the media of HepG2 and Huh7 cells treated with butyrate (Figure 4(g)). Further experiments showed that adding recombinant CXCL11 protein to conditioned media significantly enhanced NK cell migration, confirming CXCL11’s direct role in promoting NK cell migration (Figure 4(h)).

To determine whether the butyrate-induced increase in NK cell migration depends on CXCL11 re-expression, we performed a blockade assay using an anti-human CXCL11 antibody. The results showed a significant reduction in NK cell migration compared to the control (Figure 4(i)). Notably, CXCR3, the main receptor of CXCL11, is expressed on the surface of NK cell line NK-92MI.^21^ To confirm CXCL11‘s role in mediating the enhanced NK cell migration induced by media from butyrate-treated tumor cells, we added a CXCR3-neutralizing antibody to the conditioned media. Similarly, neutralizing CXCR3 on NK-92MI cells inhibited their migration toward media from butyrate-treated hepatic tumor cells, compared to the IgG control (Figure S6). These findings suggest that CXCL11 could be upregulated by butyrate in hepatic tumor cells and might contribute to the migration of NK cells.

### Butyrate alters global chromatin accessibility and cooperates with STAT4 to enhance CXCL11 transcription

**Figure 5.**
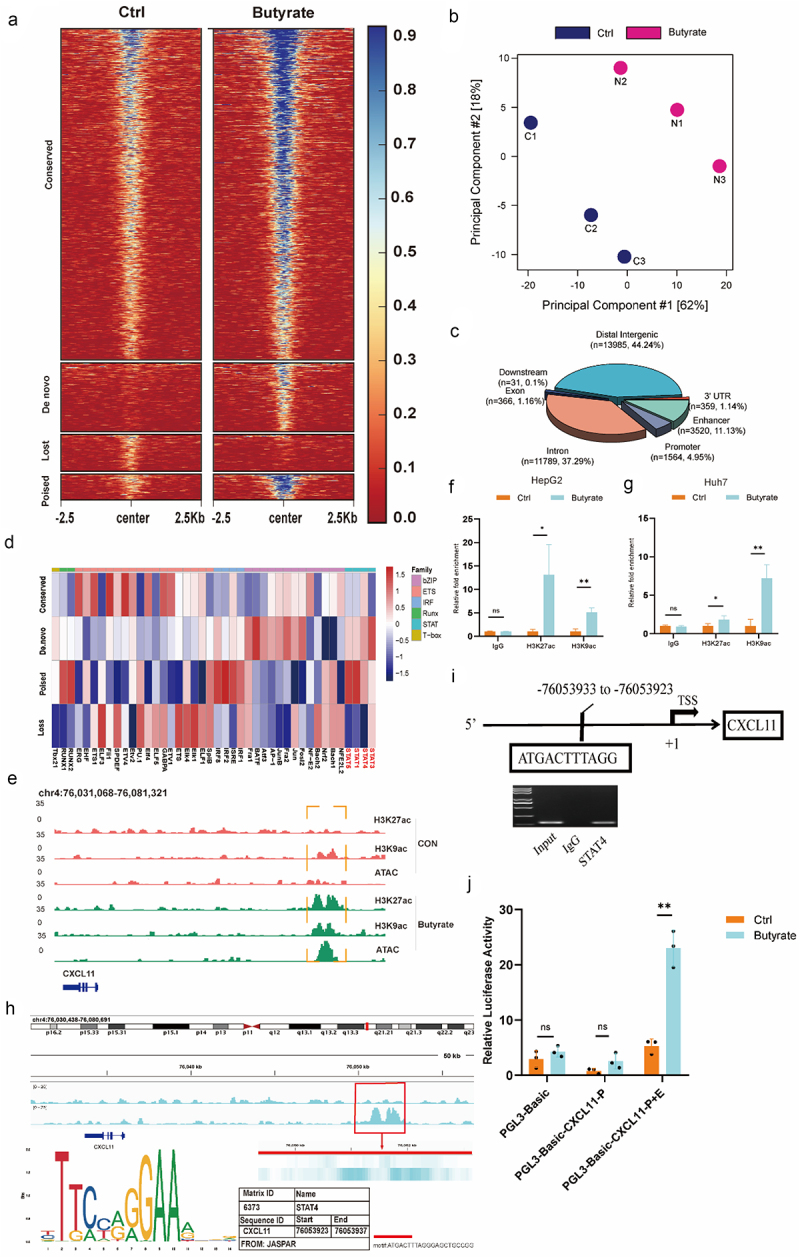
Histone modifications at gene enhancer sites modulate CXCL11 expression. (a) HepG2G2 cells were stimulated with or without butyrate for 48 h and analyzed for changes in ATAC-seq. Signal coverage heatmaps of ATAC-seq results are shown. (b) Principal component analysis (PCA) of clusters according to (a). (c) Genome browser view of normalized signals of H3K9ac, H3K27ac ChIP-seq and ATAC-seq at the CXCL11 locus in HepG2 cells with or without butyrate treatment. (d) Heatmap showing relative frequency of TF consensus motifs among five different classes defined in (a). Main TF families are highlighted at the top. Motif analysis was conducted using HOMER and visualized with R. (e) Genome browser view of normalized signals of H3K9ac, H3K27ac ChIP-seq and ATAC-seq at the locus of CXCL11 in HepG2 cells with or without butyrate treatment. (f – g) ChIP-qPCR performed in HepG2 and Huh7 cells treated with butyrate to detect the enrichment levels of CXCL11 enhancer using H3K27ac and H3K9ac antibodies and IgG control. (h) The CXCL11 enhancer region is highly enriched with H3K27ac. The sites of the putative STAT4 binding motif in the − 76053933 CXCL11 enhancer were predicted using the JASPAR database. (i) Gel electrophoresis of STAT4 enrichment at binding sites in the CXCL11 enhancer. (j) Luciferase assay of HEK293 T cells transfected for 48 h with the indicated plasmids: PGL3-basic, CXCL11 promoter and CXCL11 promoter + enhancer plasmid. Data are presented as mean ± SEM based on three independent experiments. *p < 0.05; **p < 0.01; ***p < 0.001, by two-tailed Student’s t-test.

To understand how butyrate regulates CXCL11 transcription, we analyzed changes in open chromatin regions (OCRs) and binding sites of H3K27ac/H3K9ac in hepatic tumor cells using assay for transposase-accessible chromatin sequencing (ATAC-seq)^22^ and chromatin immunoprecipitation sequencing (ChIP-seq),^23^ respectively. This analysis was conducted at intervals of 48 hours before and after butyrate stimulation (Figure 5(a) and Figure S7A). We identified four OCR clusters, representing regions that either remained unchanged or underwent specific changes during butyrate stimulation: (1) conserved: OCRs conserved with or without butyrate stimulation (*n* = 9188), (2) *De novo*: OCRs opened after 48 h butyrate stimulation (*n* = 1908), (3) poised: OCRs more selectively found after 48 h butyrate stimulation (*n* = 719), or (4) Lost: OCRs closed after 48 h butyrate stimulation (*n* = 1015). Furthermore, principal component analysis (PCA) demonstrated distinct clusters with or without butyrate stimulation (Figure 5(b)). Cluster annotations revealed that OCRs treated with butyrate were more frequent in enhancer regions than in promoter regions (Figure 5(c)). Additionally, TF motif analysis on the four OCR clusters exhibited a specific enrichment of STAT motifs (STAT1, STAT2, STAT3, STAT4) in *de novo* OCRs, whereas IRF2 (IRF) motifs were enriched in poised OCRs, and ETS motifs were enriched in the conserved and lost OCRs (Figure 5(d)). Notably, CXCL11 was located within the *de novo* OCRs cluster. Furthermore, based on the H3K27ac peaks located more than 2000 bp from the transcription start site (TSS), we identified a *de novo* enhancer near CXCL11. The enhancer of CXCL11 with reshaped chromatin accessibility partially overlapped with H3K9ac- and H3K27ac-enriched targets in butyrate-treated tumor cells (Figure 5(e)). Extensive ChIP-qPCR analysis confirmed that H3K9/H3K27 acetylation was elevated at the enhancer regions of the CXCL11 genes (Figure 5(f,g)). Further, H3K27ac ChIP-seq combined with the JASPAR database search confirmed that CXCL11 is a downstream target of STAT4 (Figure 5(h)). Gel electrophoresis revealed that STAT4 bound to the enhancer of CXCL11 more efficiently in the butyrate-treated group (Figure 5(i)). The dual-luciferase reporter assay showed that butyrate enhanced the activity of the CXCL11 enhancer, rather than its promoter (Figure 5(j)). These results underscored the role of STAT4 in regulating CXCL11 expression in butyrate-treated tumor cells.

### Butyrate-CXCL11-NK cell migration axis inhibits tumor development in vivo

**Figure 6.**
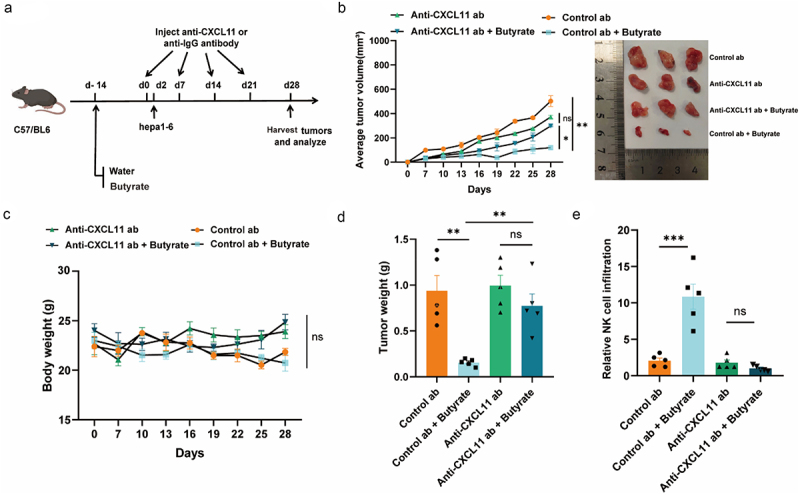
Butyrate-mediated re-expression of CXCL11 is necessary for tumor suppression in hepatic tumor mouse model. (a) Schematic representation of the experimental design for CXCL11-neutralizing assays and timeline of mouse models. (b) Average tumor sizes were monitored for the indicated conditions since tumor inoculation. *N* = 5 mice for each group. Representative images are shown at right. (c) Mice weight curves are monitored. *N* = 5 mice for each group. (d) Average tumor weight was recorded. *N* = 5 mice for each group. (e) The percentage of Hepa1–6 tumor-infiltrating NK cell was flow cytometrically measured. *N* = 5 mice for each group. Data are presented as the mean ± SEM; ns, not significant; *p < 0.05, **p < 0.01, ***p < 0.001, and ****p < 0.0001, two-tailed Student’s test.

We postulated that CXCL11 may play a crucial role in butyrate-mediated tumor suppression in a hepatic tumors mouse model. To test this hypothesis, C57BL/6 mice were given butyrate in their drinking water, subjected to tumor implantation and then injected with a CXCL11-neutralizing antibody (Figure 6(a)). Notably, we found that neutralizing CXCL11 may reduce butyrate’s tumor-inhibitory effect，indicating that CXCL11 re-expression by butyrate is essential for hepatic tumor suppression without affecting mice‘s body weight (Figure 6(b–d)).

Furthermore, cellular experiments confirmed that butyrate administration did not significantly increase NK cell infiltration into tumors in mice treated with CXCL11 antibody (Figure 6(e)) compared to control mice without CXCL11 antibody treatment. This suggests that butyrate may facilitate NK cell trafficking to tumor sites by modulating CXCL11 expression. Taken together, these findings suggest that butyrate might mediate tumor suppression by inducing CXCL11 secretion, thus enhancing NK cell recruitment to tumor sites and inhibiting tumor cell growth.

## Discussion

In our study, we metabolomically analyzed the plasma SCFA profile in hepatocellular carcinoma (HCC) patients. Our results revealed significantly decreased butyrate levels, which correlated with poor prognosis. We found that butyrate promoted CXCL11 production in tumor cells by enhancing H3K27ac and H3K9ac enrichment, thereby increasing chromatin accessibility at CXCL11 enhancer regions. This upregulation facilitated NK cell infiltration into tumors and suppressed tumor growth. Additionally, butyrate improved NK cell function both *in vitro* and *in vivo*. These findings highlight the epigenetic role of butyrate in anti-tumor immunity and suggest the potential of probiotic-based therapies for HCC patients.

Our study found a correlation between plasma SCFA concentrations and prognostic outcomes in HCC patients. A previous study showed that pathways involved in butyrate metabolism were activated in HCC patients, despite decreased plasma butyrate levels.^24^ Additionally, overexpression of genes related to butyrate metabolism was associated with a poor prognosis.^24^ These results further substantiate the findings of our research. Furthermore, liver cancer patients exhibit significantly reduced microbial diversity, especially in butyrate-producing microbes, compared to healthy individuals.^25^ Previous studies have also shown improved outcomes in colon cancer patients receiving butyrate supplementation.^24^ Thus, we speculate that changes in butyrate levels can influence liver health by modulating the intestinal microbiota, and a butyrogenic diet or butyrate-producing microbes may have therapeutic potential for HCC patients. These conclusions were based on an analysis of 36 patients, and future studies with a larger population are needed to validate these findings. Our study suggests the potential to improve outcomes in HCC patients by modulating the gut microbiota or their metabolites.

One important question arises: How dose microbiota-derived butyrate exert its anti-tumor effect? It has been reported that butyrate regulates CD8+ T cell antitumor immunity in non-small cell lung cancer (NSCLC) and colorectal cancer (CRC) patients.^24,26^ Our study also demonstrated that butyrate, a microbial metabolite, plays a pivotal role in shaping the immune landscape in an experimental HCC model, with NK cells playing a key role in butyrate-induced tumor suppression. The most significant finding was that treatment with butyrate enhanced infiltration of NK cells into the TIME. Additionally, we found that butyrate treatment improved NK cell-mediated cytotoxicity. In mice treated with antibiotics during early development, supplementation with butyrate or administration of Clostridium Butyricum promoted maturation and functional restoration of liver NK cells.^27^ These findings suggest that butyrate could act as a modulator of immune cell function. Recently, NK cell-based oncological therapy has shown promising efficacy in individuals diagnosed with advanced leukemia over the past two decades.^28^ Moreover, Adoptive Cell Transfer (ACT) therapy has been shown to be effective against hematological malignancies; however, its efficacy in most solid tumors, such as HCC, is limited due to low infiltration of NK cells within the TIME.^29^ Given butyrate’s ability to enhance both function and abundance of NK cells, leveraging gut microbiota and their metabolites for NK cell-based immunotherapy offers a promising approach for liver cancer treatment. Furthermore, due to the complexity of HCC pathogenesis and limitations of current animal models, further research using humanized models is necessary before translating these results to clinical practice.

In addition, our research illustrated that butyrate stimulated the CXCL11 secretion from tumor cells, thus potentially enhancing NK cell migration and subsequently inducing potent anti-tumor effects. The chemokines, classified as chemoattractant cytokines, play a pivotal role as mediators of leukocyte migration in immune surveillance.^30^ Increased CXCL11 expression has been associated with enhanced anti-tumor immunity in various cancers.^31,32^ While evidence suggests that CXCL11 could be a direct downstream target of butyrate and a crucial mediator of NK cell infiltration in HCC, alternative indirect regulatory mechanisms dependent on butyrate may also influence NK cell trafficking. For instance, Olink-seq shows that butyrate treatment leads to upregulation of CCL2, also known as MCP-1, compared to the untreated groups (Figure 4(e)). Interestingly, in the TIME of melanoma, inhibition of HIF-1α transcriptional activity led to the infiltration of NK cells and CD8+ T cells, which was associated with elevated release of the chemokines CCL2 and CCL5.^33^ Another notable butyrate-dependent chemokine identified by Olink-seq is MCP-3, which plays a crucial role in regulating NK cells behavior, including their migration and chemotaxis.^34^ Moreover, RNA-seq identified CX3CL1 as an adhesion molecule (Figure 4(c)). Notably, the upregulation of adhesion molecule expression was associated with increased NK cell infiltration and subsequent anti-tumor effects.^35^ Therefore, modulating adhesion molecule expression may enhance the recruitment of NK cells to the liver following butyrate treatment. Further investigations are needed to elucidate alternative regulatory mechanisms involved in butyrate-dependent NK cell trafficking.

Prior studies suggest that the anti-tumor and anti-inflammatory effects of butyrate may result from HDAC inhibition, which suppresses cell proliferation and differentiation.^36^ Moreover, active enhancers regulate gene expression by interacting with their target promoters.^37^ However, in our study, through integrated genomic analyses, we systematically examined how the enhancer landscape interact with the rapid transcriptional burst typical of hepatic tumor cells under butyrate treatment. We also proposed molecular mechanisms that regulate cascades of enhancer activation. Our genome-wide analysis revealed significant changes in chromatin accessibility and enhancer activity in hepatic tumor cell lines following butyrate stimulation. In addition to changes in enhancers, transcription factor (TF) binding was also implicated in gene expression regulation post butyrate stimulation. After 48 hours of butyrate stimulation, the enhancer region of CXCL11 was significantly activated in tumor cells, accompanied by notable enrichment of H3K9ac and H3K27ac histone modifications. JASPAR prediction and ChIP-qPCR confirmed the recruitment of STAT4 to the enhancer region, leading to transcriptional upregulation of CXCL11 and subsequent NK cell infiltration, which exerted an anti-tumor effect. This mechanistic insight not only elucidates butyrate’s molecular function but also opens new avenues for therapeutic strategies targeting NK cell-mediated antitumor response in HCC.

Due to the complexity of HCC pathogenesis and limitations of current animal models, further research utilizing humanized models is necessary before translating these results into clinical practice. Furthermore, although plasma butyrate levels were correlated with HCC patient prognosis, future studies with larger populations are necessary. It remains unknown whether different doses of butyrate can differentially regulate various immune and cancer cells in the TME. Meanwhile, determining the appropriate dose of butyrate supplementation for mice and patients remains a question to be addressed in future research.

In summary, our study suggests a positive correlation between plasma butyrate levels and prognosis in HCC patients, offering potential guidance for prognostic prediction and clinical management. Additionally, we found that butyrate could promote NK cell infiltration into tumor tissues by regulating chemotaxis through the CXCL11-STAT4 signaling pathway, highlighting a promising strategy to mitigate HCC progression and suggesting novel targets for future research and intervention.

## Supplementary Material

Supplemental Material

## Highlights

- Serum butyrate levels are decreased in patients with HCC and positively correlated with progression-free survival.
- Butyrate enhances NK cells infiltration into tumors by regulating the CXCL11-CXCR3 axis.
- Butyrate reshapes the chromatin landscape in cancer cells and induces the formation of de novo Open Chromatin Regions (OCRs).
- Histone acetylation initiates chromatin opening and facilitates STATs redeployment to de novo enhancers.
