# Supplementary material for: Gut microbial metabolite butyrate suppresses hepatocellular carcinoma growth via CXCL11-dependent enhancement of natural killer cell infiltration
Source: Gut Microbes. 2025 Jun 27;17(1):2519706. doi: 10.1080/19490976.2025.2519706 (PMC12218501; doi:10.1080/19490976.2025.2519706)
Supplement: Supplemental Material [file KGMI_A_2519706_SM3480.zip › Supplemental materials.docx]

**SUPPLEMENTARY MATERIAL**

**TABLE OF CONTENT**

[Methods and Materials 3](#_Toc185362694)

[Patient Selection/Sample Procurement 3](#_Toc185362695)

[Gas Chromatography-Mass Spectrometry (GC-MS) Analysis 3](#_Toc185362696)

[Stool Sample DNA Extraction, 16S rRNA Gene Amplification, and Bioinformatics 4](#_Toc185362697)

[Tumor immune microenvironment and Flow cytometry 4](#_Toc185362698)

[Immunofluorescence staining 5](#_Toc185362699)

[TUNEL staining 6](#_Toc185362700)

[RNA Sequencing 7](#_Toc185362701)

[Proximity ligation Assay 8](#_Toc185362702)

[Enzyme-Linked Immunosorbent Assay (ELISA) 9](#_Toc185362703)

[Real-Time qPCR 9](#_Toc185362704)

[Cell Migration Assay 9](#_Toc185362705)

[Chromatin Immunoprecipitation Sequencing (ChIP-Seq) 10](#_Toc185362706)

[Assay for Transposase Accessible Chromatin with high-throughput sequencing (ATAC-Seq) 11](#_Toc185362707)

[Cell culture 12](#_Toc185362708)

[Syngeneic Models 13](#_Toc185362709)

[NK Cell Infiltration and Flow Cytometry Analysis 13](#_Toc185362710)

[Luciferase assay 15](#_Toc185362711)

[Cell Counting Kit 8 (CCK-8) Assay 15](#_Toc185362712)

[Apoptosis assay 16](#_Toc185362713)

[EdU Assay 16](#_Toc185362714)

[LDH cytotoxic assay 17](#_Toc185362715)

[CXCR3 Neutralization and NK Cell Migration Assay 17](#_Toc185362716)

[Real-Time qPCR 18](#_Toc185362717)

[NK Cells Cytotoxicity Function and Flow Cytometry Analysis 19](#_Toc185362718)

[NK Cells Proliferation Assay 21](#_Toc185362719)

[Supplemental Figures 22](#_Toc185362720)

[Supplemental Figure Legends 27](#_Toc185362721)

[Supplemental Table 31](#_Toc185362722)

[Abbreviations 33](#_Toc185362723)

[Graphical Abstract 35](#_Toc185362724)

[Ethics declarations 36](#_Toc185362725)

[Ethics approval and consent to participate 36](#_Toc185362726)

[Consent for publication 36](#_Toc185362727)

[Availability of data and materials 36](#_Toc185362728)

[Competing interests 36](#_Toc185362729)

[Funding 36](#_Toc185362730)

[Authors’ contributions 37](#_Toc185362731)

[Acknowledgements 37](#_Toc185362732)

[Author information 37](#_Toc185362733)

[Authors and Affiliations 37](#_Toc185362734)

[Corresponding authors 39](#_Toc185362735)

[Additional information 39](#_Toc185362736)

[Publisher’s Note 39](#_Toc185362737)

# Methods and Materials

## Patient Selection/Sample Procurement

A total of 56 human peripheral blood plasma samples, from 36 HCC patients and 20 healthy liver donors, were collected at Beijing Ditan Hospital, Capital Medical University. Adult (≥ 18 years of age) patients enrolled into this study were diagnosed with HCC classified as either Barcelona Clinic Liver Cancer (BCLC) 0 – C stage or China liver cancer staging (CNLC) I - Ⅲ stage. Patients cannot be considered with severe infections or those who have received probiotics or antibiotics within the preceding month. Biological samples were collected from all patients undergoing TACE or systemic therapy prior to treatment. Fresh fecal samples from these patients were collected in sterile stool retention boxes and promptly transferred to a -80°C refrigerator for storage within 30 minutes. All samples used in this research were obtained with informed consent from the participants. This study received approval from the Capital Medical University, Beijing Ditan Hospital Ethics Committee (NO. DTEC-KY2022-046-02).

## Gas Chromatography-Mass Spectrometry (GC-MS) Analysis

Human plasma samples were thawed from the -80°C refrigerator and placed on ice until fully melted. Subsequently, 100 μL of plasma sample, 200 μL of extracting solution, and 50 μL of 50% H2SO4 were added to 1.5 mL EP tubes. After vortexing and oscillation for 10 minutes, the mixture underwent ultrasound treatment incubated in ice water for an additional 10 minutes. Centrifugation was conducted at 10,000 rpm for 15 minutes at 4°C, followed by storage at -20°C for 30 minutes. The supernatants were then transferred into 2 mL glass vials and analysed using the SHIMADZU GC2030-QP2020 NX gas chromatography-mass spectrometer[23].

## Stool Sample DNA Extraction, 16S rRNA Gene Amplification, and Bioinformatics

Stool sample DNA was extracted using the Magnetic Soil and Stool DNA Kit (TianGen, China, Catalog number: DP712). The V3-V4 region of the 16S rRNA gene was amplified using specific primers with barcodes. The resulting purified PCR product was employed for sequencing libraries, which were pooled and sequenced on NovaSeq 6000 with PE250 read sequencing, based on effective library concentration and required data amount[24].

## Tumor immune microenvironment and Flow cytometry

Mice tumor tissues were dissociated in DMEM medium (Gibco, Thermo Fisher Scientific) and digested in Collagenase IV ([Sigma-Aldrich](https://www.sigmaaldrich.com/)) containing DNase I ([Sigma-Aldrich](https://www.sigmaaldrich.com/)) on a rotor at 37°C for 40 minutes. Following digestion, the tissue was gently passed through a 200mm Cell-Strainer (BD Biosciences) in DMEM medium with 10% FBS (Gibco, Thermo Fisher Scientific) to halt the digestion process. The cell suspension was centrifuged at 500 × g for 10 minutes, and the supernatants were discarded. The cell pellets were re-suspended in 3mL red blood cell lysis buffer (Solarbio) and rested for 3 minutes at room temperature. After centrifugation at 500g for 10 minutes, the cell pellets were resuspended in the FASC buffer (PBS supplemented with 2% FBS and 100μL 0.5mM EDTA) for flow cytometric analysis using the following antibodies: FC block (BD Biosciences), BV510 (BD Biosciences), anti-Ly6G (BD Biosciences), anti-F4/80 (BD Biosciences), anti-CD3 (BD Biosciences), anti-NK1.1 (BD Biosciences), anti-B220 (BD Biosciences), anti-CD45 (BD Biosciences).

## Immunofluorescence staining

The tissue sections were deparaffinized and subsequently rehydrated in distilled water. Antigen retrieval was performed with careful attention to prevent excessive evaporation of the buffer, followed by natural cooling of the sections. Endogenous peroxidase activity was blocked by circumscribing the tissue and incubating with blocking solution. Non-specific binding was further mitigated by incubating the sections with 5% BSA for 30 minutes. The sections were then incubated with NK1.1 and CD45 (BD Biosciences) primary antibody overnight at 4°C. Following primary antibody incubation, HRP-conjugated secondary antibody was applied and incubated at room temperature for 50 minutes. The tissue was then incubated with the corresponding TSA (Tyramide Signal Amplification) dye at room temperature for 10 minutes, protected from light, and washed three times, each for 5 minutes. For antigen retrieval, sections were placed in antigen retrieval buffer and heated in a microwave at medium-low power for 8 minutes, followed by a cooling period of 8 minutes at room temperature. A second heating cycle was carried out at medium-low power for 7 minutes. Nuclei were counterstained with DAPI for 10 minutes at room temperature, protecting from light. Tissue autofluorescence was quenched using autofluorescence quenching solution B for 5 minutes, followed by extensive washing in running water for 10 minutes. Finally, the sections were mounted with a coverslip, and images were captured for subsequent analysis.

## TUNEL staining

The TUNEL (Terminal deoxynucleotidyl transferase dUTP nick end labeling) assay (Vazyme, Nanjing, China) was employed to detect apoptotic cells in paraffin-embedded tumor tissue sections. Cells with nuclear membrane collapse and nuclear disintegration were identified as apoptosis-positive. Briefly, the 4 µm thick tumor tissue sections were placed on slides and fixed with paraformaldehyde for 20 minutes, followed by three washes with PBS, each for 10 minutes. Next, 50 µl of TUNEL detection solution was applied and incubated at room temperature for 60 minutes, followed by three PBS washes. The cell nuclei were stained with DAPI (Sigma, USA). Finally, the samples were mounted with an anti-fluorescence quenching film and observed under a fluorescence microscope (Olympus, Japan).

## RNA Sequencing

Total RNA was extracted from HepG2 cells and HepG2 cells treated with 1mM Butyrate for 48 hours, using TRIzol Reagent (Life technologies). RNA integrity was assessed using the RNA Nano 6000 Assay Kit of the Bioanalyzer 2100 system (Agilent Technologies, CA, USA). After purification with the AMPure XP system (Beckman Coulter, Beverly, USA), cDNA fragments of 370~420bp were used for library construction. Cluster generation was performed on a cBot Cluster Generation System using TruSeq PE Cluster Kit v3-cBot-HS (Illumina) according to the manufacturer’s instructions. The library preparations were then sequenced on an Illumina Novaseq platform, generating 150bp paired-end reads. Differential expression analysis of the two groups was performed using the DESeq2 R package (1.20.0). The resulting P-values were adjusted using the Benjamini and Hochberg’s approach for controlling the false discovery rate. Genes with an adjusted P-value <=0.05 found by DESeq2 were assigned as differentially expressed.

## Proximity ligation Assay

The cellular supernatant of HepG2 cells and HepG2 cells treated with Butyrate was collected, and the proteomic profile was analysed using the Olink Target 96 inflammation panels (Olink Proteomics, Uppsala, Sweden). The Proximity Extension Assay (PEA) technology behind the Olink panels was utilised and then quantified by quantitative PCR. Briefly, during the incubation step, the 92 antibody pairs labelled with DNA oligonucleotides were bound to their target protein in the samples. Subsequently, oligonucleotides extended in proximity to each other and were amplified by quantitative PCR. The protein expression levels were ultimately generated using an arbitrary unit called Normalized Protein eXpression (NPX). NPX is derived from the Ct values obtained from the qPCR using extension control, inter-plate control, and a correction factor. NPX measurements below the lowest level of detection for each marker were set to the limit of detection (LOD) value. Log2 fold changes and associated p-values were calculated by Student’s t-test, with p-values less than 0.05 considered statistically significant.

## Enzyme-Linked Immunosorbent Assay (ELISA)

Enzyme-Linked Immunosorbent Assay (ELISA) was conducted in accordance with the manufacturer’s protocol. The concentrations of CXCL11 (Human) in the culture supernatant of Butyrate-treated cells were measured using a commercialised ELISA kit (Invitrogen).

## Real-Time qPCR

After 48h stimulation, total RNAs were extracted from HepG2 or Huh7 cells using TRIzol Reagent (Life technologies). 2µg of RNA was synthesized for cDNA using (Vazyme Biotech) following manufacturer provided protocol and 100ng cDNA was utilised for RT-qPCR on ABI Prism 7500 (Applied Biosystems) with the following primers: human CXCL11, 5’- GACGCTGTCTTTGCATAGGC -3’ and 5’- GGATTTAGGCATCGTTGTCCTTT-3’. The mean CT values of target genes were normalised to endogenous Actin, and comparisons were made using the 2-ΔΔCt method. All experiments were performed at least in triplicate, and the relative mRNA levels of Butyrate-treated samples, compared to that of control samples, are presented.

## Cell Migration Assay

NK cell migration was carried out as previously described with minor modifications [25]. In brief, HepG2 and Huh7 cells were treated with Butyrate or PBS control for 48 hours in Reduced-serum medium. After 48 hours, cell culture supernatants were harvested and concentrated using Merck Centricon tubes (3kDa) at 5000g for 30 minutes at 4°C. Subsequently, 2.5 x 10^5 NK cells (NK-92MI) were placed in the upper chamber of a 24-well Transwell culture plate (Corning). Simultaneously, 600μL of concentrated culture supernatants were collected and added to the bottom chamber of the Corning Transwell system. The CXCL11 neutralising antibody was added to the conditioned media. Reduced-serum medium added with Butyrate served as a negative control. The Transwell culture plates were incubated at 37°C for 4 hours, after which migrated cells in the bottom chamber were harvested into 1.5mL EP tubes. Subsequently, 50μL of fluorescent Count Bright Absolute Counting Beads (Invitrogen) were added to the fluorescence-activated cell sorting (FACS) tubes, and cells were resuspended in a fixed volume of 250μL PBS. Data were collected using a BD canto flow cytometer and analysed with FlowJo software.

## Chromatin Immunoprecipitation Sequencing (ChIP-Seq)

HepG2 cells treated with Butyrate (Sigma-Aldrich) or PBS control were collected, and approximately 5 x 10^7 cells each underwent ChIP. Initially, cells underwent chemical cross-linking with formaldehyde (1% final concentration), followed by glycine treatment (125mM) for 10 minutes each. Subsequently, nuclear lysis using nuclear lysis buffer was performed, and DNA was fragmented through ultrasonification (diagenode). Immunoprecipitation was carried out with magnetic beads using antibodies for H3K27ac (Abcam), STAT4(Cell signaling), H3K4me3(Cell signaling), or H3K9ac (Cell signaling) with appropriate isotype controls (Cell signaling). Immunoprecipitated beads were collected, and ChIP DNA was purified for library generation (NEB Next Ultra II DNA Library Prep Kit for Illumina) according to the manufacturer’s instructions. Sequencing was conducted on the Illumina HiSeq 2500 or Genome Analyzer II platform. ChIP-qPCR was performed using following primers: human CXCL11, 5’- AGCACACTGTCACCTCTCAA -3’ and 5’- AGCCTGCAGAATCTGTCTCA -3’; human STAT4, 5’- ATGACTTTAGGGAGCTGCCGG -3’ and 5’- CCGGCAGCTCCCTAAAGTCAT -3’ and enrichment was compared with IgG-precipitated DNA.

## Assay for Transposase Accessible Chromatin with high-throughput sequencing (ATAC-Seq)

ATAC-seq was conducted as previously described with minor modifications. In brief, 50,000 cells were counted and washed with cold DPBS, then centrifuged at 500 x g for 5 minutes at 4°C. The cells were treated with resuspension buffer (RSB) (10 mM Tris-HCl, pH 7.4, 3mM MgCl2, 10mM NaCl) supplemented with 0.1% NP40, 0.01% digitonin, and 0.1% Tween-20. After incubating the samples on ice for 3 minutes, they were washed with 1 ml cold RSB containing 0.1% Tween-20, then centrifuged at 500 x g for 10 minutes at 4°C. The sediment was resuspended in 50 μl transposition mix (25 μl 2 x TD buffer, 2 μl transposase, 0.5 μl 10% Tween-20, 0.5 μl 1% digitonin, 16.5 μl PBS, 3.5 μl RNA-free water) and incubated at 37°C with shaking at 1000 rpm for 30 minutes. The fragmented DNAs were then purified with AmpureXP beads and sequenced using an Illumina.

## Cell culture

The mouse hepatic tumor cell line Hepa1-6, human hepatic tumor cell line (HepG2 and Huh7) and 293T cells were procured from the American Type Culture Collection (ATCC) and cultured in Dulbecco's Modified Eagle's Medium (DMEM; Gibco, Thermo Fisher Scientific). NK-92MI cells were sourced from ATCC (MEM; Gibco; C12571500BT) and cultivated in Alpha-Minimum Essential Medium (Gibco, Thermo Fisher Scientific), 12.5% FBS (Gibco, Thermo Fisher Scientific), 100 units/mL of penicillin (Gibco, Thermo Fisher Scientific), 100 µg/mL of streptomycin (Gibco, Thermo Fisher Scientific), 0.2 mM inositol ([Sigma-Aldrich](https://www.sigmaaldrich.com/)), 0.02 mM folic acid ([Sigma-Aldrich](https://www.sigmaaldrich.com/)) and 0.1 mM 2-mercaptoethanol (Gibco, Thermo Fisher Scientific).

## Syngeneic Models

Subcutaneous injection of 2 x 10^5 Hepa1-6 cells into the right buttock of C57BL/6 mice was performed. At 14 days post-injection, mice were subjected to treatment with 30 mM sodium butyrate ([Sigma-Aldrich](https://www.sigmaaldrich.com/)) in drinking water until the conclusion of the experiment. To minimize potential bias from ad libitum intake, we housed only 1-2 mice per cage and precisely measured the daily consumption of each individual mouse. Peripheral blood and feces from all mice were collected and subjected to targeted short-chain fatty acid profiling. Depletion of NK cells was achieved by intraperitoneal injection of 50 μg anti-mouse polyclonal Ultra-LEAF Purified anti-Asialo-GM1 antibodies (BioLegend) once every week for 4 weeks. Ultra-LEAF purified mouse IgG1, κ-isotype control antibody (BioLegend) served as the isotype control.

For in vivo CXCL11 neutralization, 100 μg of CXCL11 antibodies (Invitrogen) were injected intraperitoneally into C57BL/6 mice once every week for 4 weeks. Ultra-LEAF purified mouse IgG1, κ-isotype control antibodies (BioLegend) were used as the isotype control.

## NK Cell Infiltration and Flow Cytometry Analysis

Tumor tissues from mice were dissociated in DMEM medium and digested in Collagenase IV containing DNase I on a rotor at 37°C for 40 minutes. Following digestion, the tissue was gently ground through a 200 mm Cell-Strainer in DMEM medium (Gibco) with 10% FBS to halt the digestion. The cell suspension was centrifuged at 500 × g for 10 minutes, and after discarding the supernatants, cell pellets were re-suspended in 3 mL red blood cell lysis buffer and left for 3 minutes at room temperature. After centrifugation at 500g for 10 minutes, the cell pellets were re-suspended in the FACS buffer (PBS supplemented with 2% FBS and 100 μL 0.5 mM EDTA) for flow cytometric analysis using the following antibodies: FC block (BD Biosciences), BV510 (BD Biosciences), NKp46 (BD Biosciences), anti-CD3 (BD Biosciences), anti-NK1.1 (BD Biosciences). Data were collected using a BD Fortessa flow cytometer and analyzed with FlowJo software.

Mice spleens were harvested and were gently ground through the 200mm Cell-Strainer. Then cell suspension was added to a 15-ml centrifuge tube and centrifugated at 1500rpm for 7min. The supernatants were discarded and cell pellets were re-suspended by 3mL red blood cell lysis buffer and rest for 3min at room temperature. which was allowed to rest at room temperature for 5min at 1500 RPM for 7min. After centrifuged at 500g for 10 min, the cell pellets were resuspended in the FASC buffer (PBS supplemented with 2% FBS and 100μL 0.5mM EDTA) for flow cytometric analysis using the following antibodies: FC block (BD Biosciences), BV510 (BD Biosciences), NKp46 (BD Biosciences), anti-CD3 (BD Biosciences), anti-NK1.1 (BD Biosciences). Data were collected using a BD Fortessa flow cytometer and analyzed with FlowJo software.

## Luciferase assay

HEK293 cells were co-transfected with PGL3-basic-luc, PGL3-basic-CXCL11-Promoter plasmid, PGL3-basic-CXCL11-Promoter + Enhancer plasmid after transfection for 6 h, the cells were induced with or without butyrate for 48 h, and lysed for 15 min on ice. The lysate was analyzed using the Dual-Luciferase Reporter Assay System (Beyotime Biotechnology). Results are presented as the average of three measurements.

## Cell Counting Kit 8 (CCK-8) Assay

Cell viability was assessed using the Cell Counting Kit 8 (CCK-8, Vazyme) assay, following the provided instructions. Transfected cells were seeded in a 96-well plate at a density of 5 × 10^4 cells and cultured for 48 hours. Subsequently, 10 μl of CCK-8 solution was added to each well, and the plate was incubated for 1 hour at 37 °C. Absorbance was measured at 450 nm using Vector 5 (Bio-Tech Instruments, USA). The CCK-8 assay was employed to examine the impact of sodium butyrate intervention on the growth and proliferation of liver cancer cells at various concentrations. The inhibitory concentrations of 50% proliferation (IC50) of sodium butyrate were calculated using GraphPad Prism software version 5.0. The experiment was conducted in triplicate.

## Apoptosis assay

To determine the apoptotic rate, liver cancer cells were seeded into six-well plates. The Annexin V-PE/7-AAD Apoptosis Detection Kit (Vazyme) was utilised according to the manufacturer’s instructions. Briefly, cells were washed twice in ice-cold PBS, incubated with 100 μl 1 × Binding Buffer supplemented with 5 μl Annexin V-PE and 5 μl 7-AAD Staining Solution. After 10 minutes, an additional 400 μl 1 × Binding Buffer was added. The apoptotic rate was measured by flow cytometry, and data were analysed using FlowJo software.

## EdU Assay

For the EdU staining, the EdU Cell Proliferation Kit (Beyotime) was employed to assess the proliferation of HepG2 and Huh7 cells treated with Butyrate following the manufacturer’s instructions. Cells were incubated in culture medium containing 10 μM EdU reagent at 37°C for 2 hours. Subsequently, the samples were collected, and the nuclear envelope was disrupted with FOXP3 (Tonbo Biosciences). Pellets were stained with the reaction solution. Data were collected and analysed using a BD canto flow cytometer and FlowJo software.

## LDH cytotoxic assay

HepG2 and Huh7 cells were treated with Butyrate for 48 hours. NK cells (NK92MI) served as effector cells and were incubated with HepG2 and Huh7 cells at an effector-to-target ratio of 1:1, 10:1, 20:1 in 96-well round bottom culture plates. Cytotoxicity was assessed using the lactate dehydrogenase (LDH) kit (Beyotime) following the manufacturer's instructions.

## CXCR3 Neutralization and NK Cell Migration Assay

HepG2 and Huh7 cells were treated with Butyrate or PBS control for 48h in Reduced-serum medium. After 48h, cell culture supernatants were harvested and concentrated using Merk Centricon tubes(3kDa) at 5000g for 30min at 4 °C. And then 2.5 x 10^5 NK cells (NK-92MI) were placed in the upper chamber of a 24-well Transwell culture plates (Corning). Meanwhile, 600μL concentrated culture supernatants were collected and added in the bottom chamber of the Corning Transwell system. And CXCR3 neutralizing antibody (Selleck) were added to the conditioned media. Reduced-serum medium added Butyrate served as a negative control. The Transwell culture plates was incubated at 37 °C for 4 h, after which migrated cells in the bottom chamber were harvested into the 1.5mL EP tubes. Then 50μL fluorescent Count Bright Absolute Counting Beads (Invitrogen) were added to the fluorescence-activated cell sorting (FACS) tubes, and cells were resuspended in a fixed volume of 250μL PBS. Data were collected using a BD canto flow cytometer and analyzed with FlowJo software.

## Real-Time qPCR

After 48h stimulation, total RNAs were extracted from HepG2 or Huh7 cells using TRIzol Reagent (Life technologies). 2µg of RNA was synthesized for cDNA using (Vazyme Biotech) following manufacturer provided protocol and 100ng cDNA was used for RT-qPCR on ABI Prism 7500 (Applied Biosystems) with the following primers: human ULBP1, 5’-CCACCAGGACTGGCAAACTG-3’ and 5’-ATTGGGAGGCCAAGGTGGTA-3’; human ULBP2, 5’-CAGGCACAACCCAACTCAGG-3’ and 5’-GCCAGACAGAAGGGCGAGTT-3’; human ULBP3, 5’-CCTCGCGATTCTTCCGTACC-3’ and 5’-GCCAGACAGAAGGGCGAGTT-3’; human ULBP4, 5’-TCGCCACCAATGGAGAGAAA-3’ and 5’-TCGCCACCAATGGAGAGAAA-3; human ULBP5, 5’-GCTTCTGCTCCTGCTGTCCA-3’ and 5’-GGGACTGACGGGTGTGACTG-3’; human ULBP6, 5’-GCCATGTCCTCAGGCACAAC-3’ and 5’-TCAGATGCCAGGGAGGATGA-3; human MICA, 5’-CCTGCAATCCCAGCACTTTG-3’ and 5’-ATTCACCACCAAGCCCGTCT-3’; human MICB, 5’-CACGTTCGCCCTTTGTTCAG-3’ and 5’-GGAGGCAGAGGTTGCAGTGA-3’; human CD112, 5’-ACGGTCACCTGCAAAGTGGA-3’ and 5’-ACGGCCGAGGTACCAGTTGT-3’; human CD155, 5’-TGTCCCGTAACGCCATCATC-3’ and 5’-CCAAAGGACCTCACGGGAAC-3’. The mean CT values of target genes was normalized to endogenous Actin and comparisons were performed by using the 2-ΔΔCt method. All experiments were performed at least biological triplicate and the relative mRNA levels of Butyrate treated samples, which were compared to that of control samples were shown.

## NK Cells Cytotoxicity Function and Flow Cytometry Analysis

For NK cytotoxicity function in vivo, mice spleens were harvested and were gently ground through the 200mm Cell-Strainer. Then cell suspension was added to a 15-ml centrifuge tube and centrifugated at 1500rpm for 7min. The supernatants were discarded and cell pellets were re-suspended by 3mL red blood cell lysis buffer and rest for 3min at room temperature and then centrifuged at 1500rpm for 7min. The cell pellets were resuspended in the FASC buffer (PBS supplemented with 2% FBS and 100μL 0.5mM EDTA) for flow cytometric analysis. The freshly isolated cells were stimulated with PMA (100 ng/ml) (Sigma-Aldrich) and ionomycin (500ng/ml) (Sigma-Aldrich) for 4 h. For CD107a (BD Biosciences) staining, cells were incubated with CD107a antibody for 4 h. Following surface staining, cells were fixed with intracellular fixation buffer for a duration of 20 minutes, followed by permeabilization using permeabilization buffer for a period of 10 minutes. Subsequently, intracellular staining was conducted utilizing IFN-γ (BD Biosciences) antibodies diluted in the permeabilization buffer. Data were collected using a BD Fortessa flow cytometer and analyzed with FlowJo software.

For NK cytotoxicity function in vitro, the NK92MI cells were treated with butyrate for 48h. After centrifuged at 500g for 10 min, the cell pellets were resuspended in the FASC buffer (PBS supplemented with 2% FBS and 100μL 0.5mM EDTA) for flow cytometric analysis. Cells were fixed with intracellular fixation buffer for a duration of 20 minutes, followed by permeabilization using permeabilization buffer for a period of 10 minutes. Subsequently, intracellular staining was conducted utilizing IFN-γ (BD Biosciences) and TNF-α (BD Biosciences) antibodies diluted in the permeabilization buffer. Data were collected using a BD Fortessa flow cytometer and analyzed with FlowJo software.

## NK Cells Proliferation Assay

NK92 cell lines stably transduced to express GFP were seeded into the 96 well plate (~20000 cells seeded per well) with or without butyrate stimulation. Cell proliferation was measured by GFP real time imaging using an IncuCyte ZOOM (Essen) for 5 days.

# Abbreviations

***ACT:*** Adoptive Cell Transfer Therapy

***CCK-8:*** Cell Counting Kit 8

***CRC:*** Colorectal cancer

***Elisa:*** Enzyme-linked immunosorbent assay

***GC-MC:*** Gas chromatography-mass spectrometry

***GPCR:*** G-protein-coupled receptor

***GSEA:*** Gene set enrichment analysis

***HCC:*** Hepatocellular carcinoma

***HD:*** Healthy donors

***HDACi:*** Histone deacetylase inhibitor

***IHC:*** Immunohistochemistry

***INSO:*** Ionomycin

***LDH:*** Lactate dehydrogenase

***LEfSe:*** Linear discriminant analysis effect size

***LOD:*** Limit of detection

***NK cells:*** Natural killer cells

***NKG2D:*** Natural killer group 2D

***NMDS:*** Non-metric multidimensional scaling

***NPX:*** Normalized Protein eXpression

***NSCLC:*** non-small cell lung cancer

***OCRs:*** Open chromatin regions

***OPLS:*** Orthogonal partial least squares-discriminant

***PCA:*** Principal Component Analysis

***PEA:*** Proximity Extension Assay

***PFS:*** Progression free survival

***PMA:*** Phorbol 12-Myristate 13-Acetate

***RSB:*** Resuspension buffer

***SCFAs:*** Short chain fatty acid

***scRNA-seq:*** Single-cell sequencing

***STAT4***: Signal transducer and activator of transcription 4

***TIME:*** Tumor immune microenvironment

***TNF:*** Tumor necrosis factor

***TSNE:*** T-distributed Stochastic Neighbor Embedding

***UMAP :*** Uniform manifold approximation and projection

# Ethics declarations

# Ethics approval and consent to participate

The studies involving human participants were reviewed and approved by the ethics committee of Beijing Ditan Hospital. The patients/participants provided their written informed consent to participate in this study. And all animal studies were approved by the Animal Research Ethics Committee of Beijing Ditan Hospital.

## Consent for publication

Not applicable.

## Availability of data and materials

The datasets used and/or analyzed during the current study are available from the corresponding author on reasonable request.

## Competing interests

The authors declare that they have no competing interests.

**Funding**

This study was supported in part by grants from the National Key R&D Program of China (2022YFC2304500), Ministry of Science and Technology of People’s Republic of China (grant# 2023YFC2306003), the Beijing Municipal of Science and Technology Major Project (Z221100007422002), National Key R&D Program of China (2021YFC2301801), the Capital Funds for Health Improvement and Research (CFH-2024-1-2181), Beijing igandan foundation (iGandanF-1082023-GSH011, the National Natural Science Foundation of China (grant# 32270635), National Natural Science Foundation of Beijing Municipality (No. 7232082), and Beijing Research Center for Respiratory Infectious Diseases Project (BJRID2024-010).

## Authors’ contributions

MHZ, XFH, YLZ, XW and HCX conceived and designed this study; MHZ and XFH performed the experiments; MHZ, XFH and YLZ analyzed the data; MHZ and YLZ organized the figures and drafted the initial manuscript; MHZ, XXY, MHY, XW and HCX revised this manuscript; all authors read and approved the final manuscript.

# Acknowledgements

## Author information

Menghan Zhang, Xuefeng Huang and Yanlong Zhang contributed equally to this work.

## Authors and Affiliations

Center of Liver Diseases Division 3, Beijing Ditan Hospital, Capital Medical University, 8 Jingshundong Street, Chaoyang District, Beijing 100015, China

Menghan Zhang, Yifan Xu, Lei Ma & Huichun Xing.

Beijing Key Laboratory of Emerging Infectious Diseases, Institute of Infectious Diseases, Beijing Ditan Hospital, Capital Medical University, Beijing 100015, China

Xuefeng Huang, Yanlong Zhang, Minghang Yu, Xiaoxue Yuan & Xi Wang

Beijing Institute of Infectious Diseases, Beijing, 100015, China

Xuefeng Huang, Minghang Yu, Xiaoxue Yuan & Xi Wang

National Center for Infectious Diseases, Beijing Ditan Hospital, Capital Medical University, Beijing 100015, China

Xuefeng Huang, Minghang Yu, Xiaoxue Yuan & Xi Wang

National Key Laboratory of Intelligent Tracking and Forecasting for Infectious Diseases, Beijing,100015, China

Xuefeng Huang, Minghang Yu, Xiaoxue Yuan & Xi Wang

Department of Oncology, Capital Medical University, Beijing, 100069, China

Xuefeng Huang, Minghang Yu & Xi Wang

Shanxi Bethune Hospital, Shanxi Academy of Medical Sciences, Tongji Shanxi Hospital, Third Hospital of Shanxi Medical University, Taiyuan, China.

Yanlong Zhang

Peking University Ditan Teaching Hospital, Beijing 100015, China

Huichun Xing

## Corresponding authors

Correspondence to Huichun Xing and Xi Wang.

# Additional information

# Publisher’s Note

Springer Nature remains neutral with regard to jurisdictional claims in published maps and institutional affiliations.

# Supplemental Table

**Supplemental Table S1**

**Clinical Characteristics of the Enrolled Participants in the Study.**

| **Clinical and pathological indexes** | **Healthy (n = 20)** | **HCC (n = 36)** |
| --- | --- | --- |
| **Age(y), mean (SD)** | 56.05 ± 5.27 | 57.56 ± 7.12 |
| **Male, n (%)** | 9 (45%) | 20 (55.56%) |
| **BMI, (kg/m2)** | 23.92 ± 2.76 | 23.42 ± 1.68 |
| **Previous history, n (%)** |  |  |
| Diabetes | 3 (15%) | 4 (11.11%) |
| Hypertention | 4 (20%) | 6 (16.67%) |
| Coronary heart disease | 5 (25%) | 6 (16.67%) |
| **AFP (ng/ml), n (%)** |  |  |
| ≤20 | - | 16 (44.44%) |
| ＞20 | - | 20 (55.56%) |
| **Tumor size (cm), n (%)** |  |  |
| ≤5 | - | 29 (80.56%) |
| ＞5 | - | 7 (19.44%) |
| **Tumor number, n (%)** |  |  |
| ＜2 | - | 34 (94.45%） |
| ≥2 | - | 2 (5.55%） |
| **Child-Pugh, n (%)** |  |  |
| A | - | 30 (83.33%) |
| B | - | 6 (16.67%) |
| **BCLC stage** |  |  |
| BCLC 0/A | - | 32 (88.89%) |
| BCLC B/C | - | 4 (11.11%) |
| **ALT (U/L)** | 19.51 ± 9.64 | 65.10 ± 60.51 ******* |
| **AST (U/L)** | 20.53 ± 4.09 | 67.49 ± 68.68 ******* |
| **ALP (U/L)** | 61.55 ± 10.88 | 137.80 ± 168.20 * |
| **Albumin (g/L)** | 46.99 ± 2.04 | 35.53 ± 13.41 *** |
| **CHE** | 8027 ± 1088 | 4006 ± 2190 *** |
| **Total bilirubin (μmol/L)** | 13.49 ± 3.87 | 38.74 ± 36.50 ** |
| **Direct bilirubin (μmol/L)** | 4.27 ± 1.35 | 16.43 ± 21.86 ** |
| **Creatinine (μmol/L)** | 78.12 ± 11.43 | 80.25 ± 27.16 |
| **INR** | 1.05 ± 0.05 | 1.39 ± 0.33 *** |
|  |  |  |

**Supplemental Table S2**

**Univariate analysis.**

**Supplemental Table S3**

**Multivariate analysis.**

# Supplemental Figure Legends

Figure S1. The difference of plasma short-chain fatty acid (SCFAs) and fecal microbiota between HD and HCC patients. (A) Bacterial alpha diversity metrics (Chao1 abundance estimator, Simpson and Shannon diversity index) for HD and HCC groups. The y-axis represents Chao1 and Shannon index on the OTU level. ****p*<0.001, by Mann-Whitney test. (B) Bacterial beta diversity metrics metrics，Non metric multidimensional representation (NMDS) of fecal microbiota composition between HCC patients(red) and HD (blue) (stress: 0.138). Circles represent confidence interval of 95%. *** *p* < 0.001, by Mann Whitney test. (C) LEfSe (Linear discriminant analysis effect size) on the fecal microbiome shows the differences in taxa between HD (green) and HCC (red) groups (from phylum to genus levels). (D) The percentage of butyrate-producing bacteria on the genus level from 16S rRNA sequencing of feces from HD and HCC groups. (E) Orthogonal projections to latent structures-discriminant analysis (OPLS-DA) score plots show a significant difference in peripheral vein plasma samples between HD and HCC groups. Health donors are shown in blue, and HCC patients are shown in red, x-axis and y-axis represent the first 2 principal components (PC1 and PC2). (F) The plasma level of SCFAs in HD and HCC. ns, not significant; **p* < 0.05, ***p* < 0.01, ****p* < 0.001, and *****p* < 0.0001.

Figure S2. The concentration of butyrate in both blood serum and colon contents was analyzed.

Figure S3. Representative images of TUNEL stainings of tumor sections before and after butyrate treatment(green, TUNEL positive; red, CD45 positive; blue, DAPI). Scale bar = 50 um.

Figure S4. Butyrate stimulation increases expression of NK cell ligands and the susceptibility of HepG2 and Huh7 cells to NK lysis. (A) The spleen single-cell suspensions were stimulated with PMA and Ionomycin in vitro. IFN-γ^+^ and CD107A^+^ NK cells were analyzed by flow cytometry. Representative figures and bar graph are shown. (B) HepG2 and Huh7 cells were treated with either PBS or the butyrate(1mM) for 48h. The mRNA expression of the indicated NK cell ligands was measured by RT-qPCR and compared to PBS-treated cells. (C) Cytotoxicity of NK cells against HepG2 and Huh7 cell lines was measured at different E:T ratios with a 4h LDH-release assay. HepG2 and Huh7 cell lines were stimulated with 1 mM butyrate for 48 h, then washed and used as target cells. (D) Changes in fluorescence intensity during NK92-MI cell line proliferation assays using IncuCyte Zoom imaging system and image processing software. Representative figures and proliferation curves are shown. (E) The NK92-MI cell line was stimulated with PMA and Ionomycin. IFN-γ^+^ and TNF-α^+^ cells were analyzed by flow cytometry. Representative figures and bar graph are shown.

Figure S5. Butyrate suppresses the cell growth of hepatic tumor cell lines. (A). Cells proliferation assay of HepG2 and Huh7 cell after butyrate treatment at 1, 2.5, 5, 10, 20, 40 and 80 mM for 48h by CCK-8 assay. (B) Apoptosis assay of HepG2 and Huh7 cells after butyrate treatment at 0, 1, 2.5 and 5mM for 48h by flow cytometer. Quantification of apoptosis-positive cells are shown in right. (C) The mean fluorescence intensity (MFI) of HepG2 and Huh7 cells after butyrate treatment at 0, 1, 2.5 and 5mM for 48 h by flow cytometer. The quantification of percentage of EdU - positive cells of HepG2 and Huh7 cells was shown by bar graph. Data are shown as Mean ± SD of three independent experiments. ns, not significant; *P < 0.05, **P < 0.01, ***P < 0.001, and ****P < 0.0001.

Figure S6. Average number of NK cells that migrated toward conditioned media collected from HepG2 and Huh7 cells that pretreated with butyrate, with or without CXCR3-neutralizing antibodies. Data are presented as mean ± SD; *P < 0.05, **P < 0.01, and ***P < 0.001.

Figure S7. HepG2 cells were stimulated with or without butyrate stimulation for 48 h, and analyzed for changes in H3K9ac, and H3K27ac binding by ChIP-seq. Signal coverage heatmaps of H3K9ac H3K27ac ChIP-seq were shown.
