# Supplementary figures and images for: Gut microbial metabolite butyrate suppresses hepatocellular carcinoma growth via CXCL11-dependent enhancement of natural killer cell infiltration
Source: Gut Microbes. 2025 Jun 27;17(1):2519706. doi: 10.1080/19490976.2025.2519706 (PMC12218501; doi:10.1080/19490976.2025.2519706)

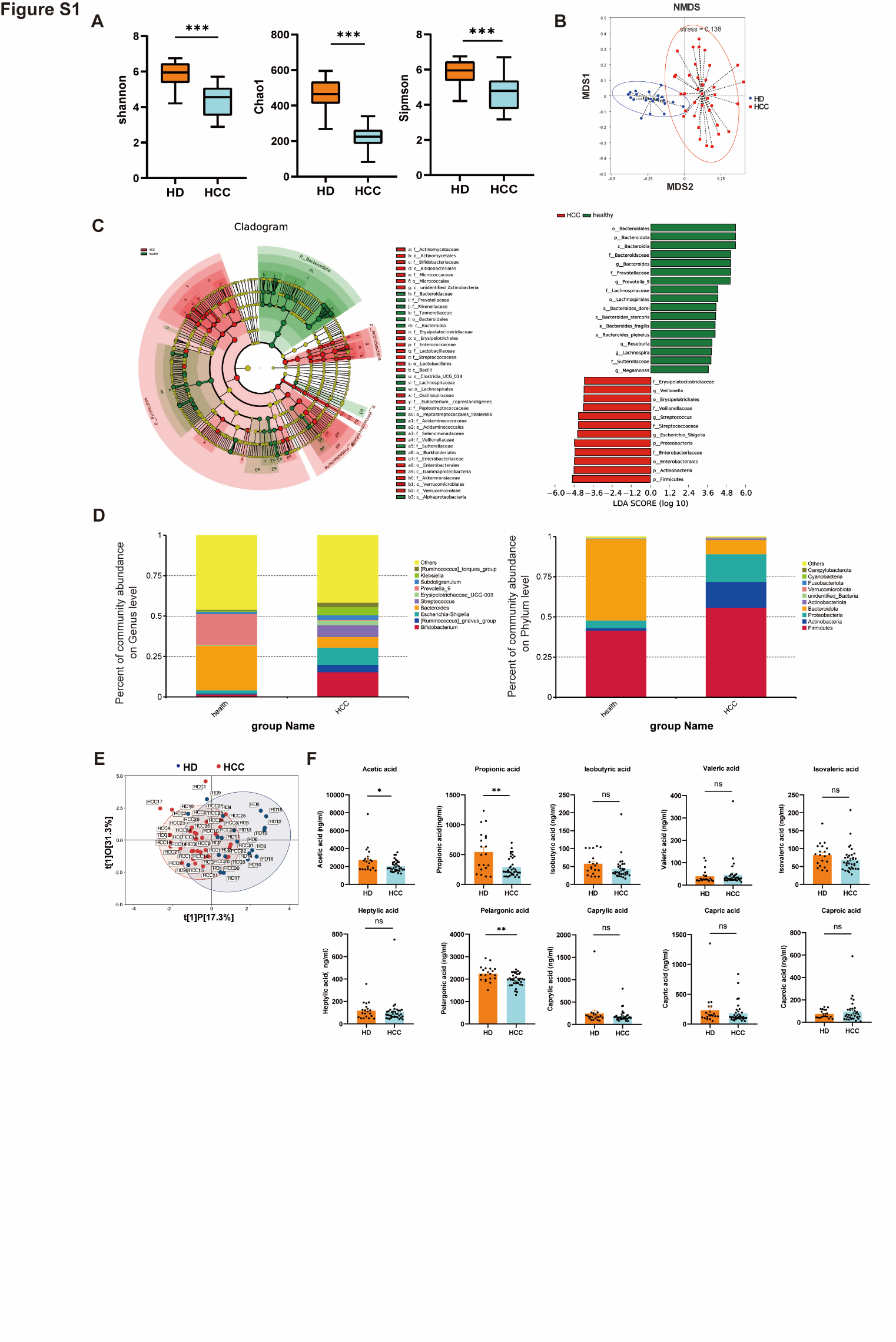


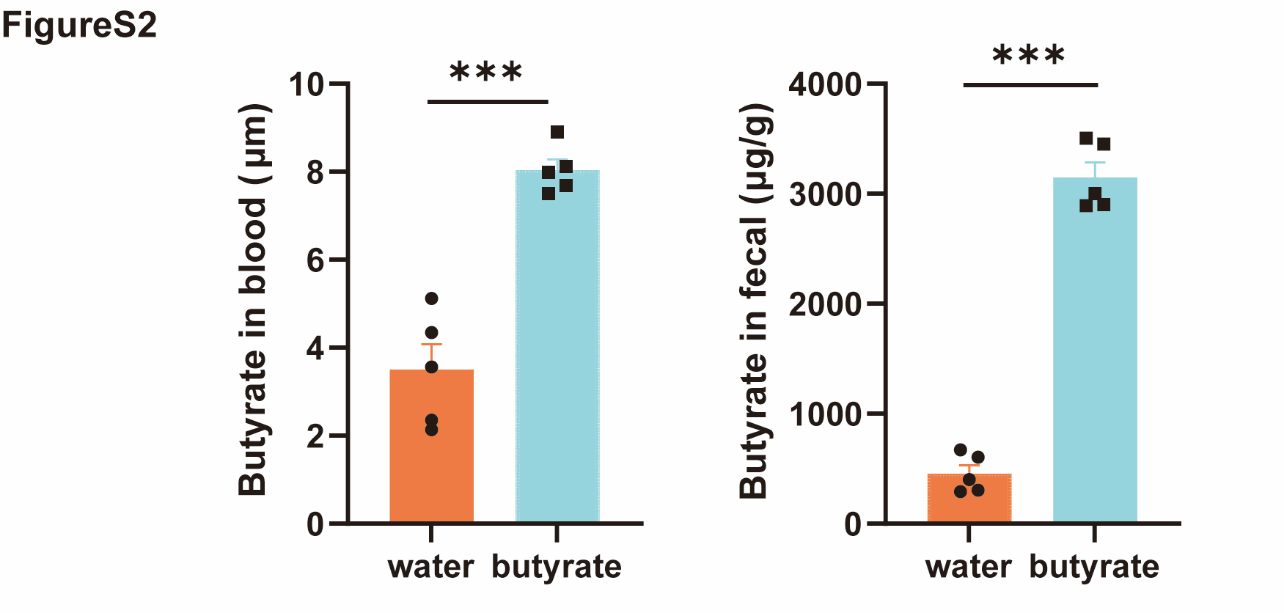


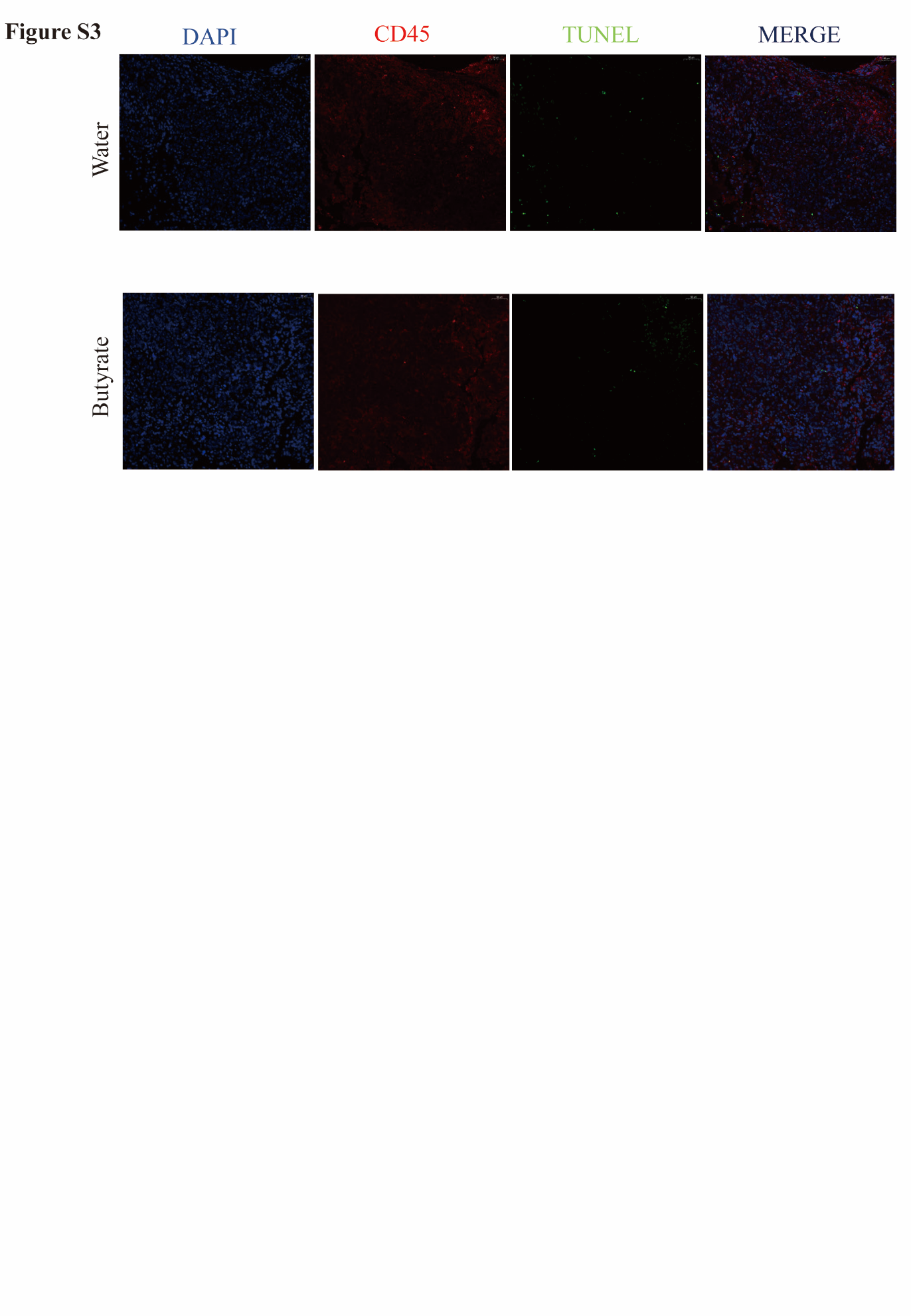


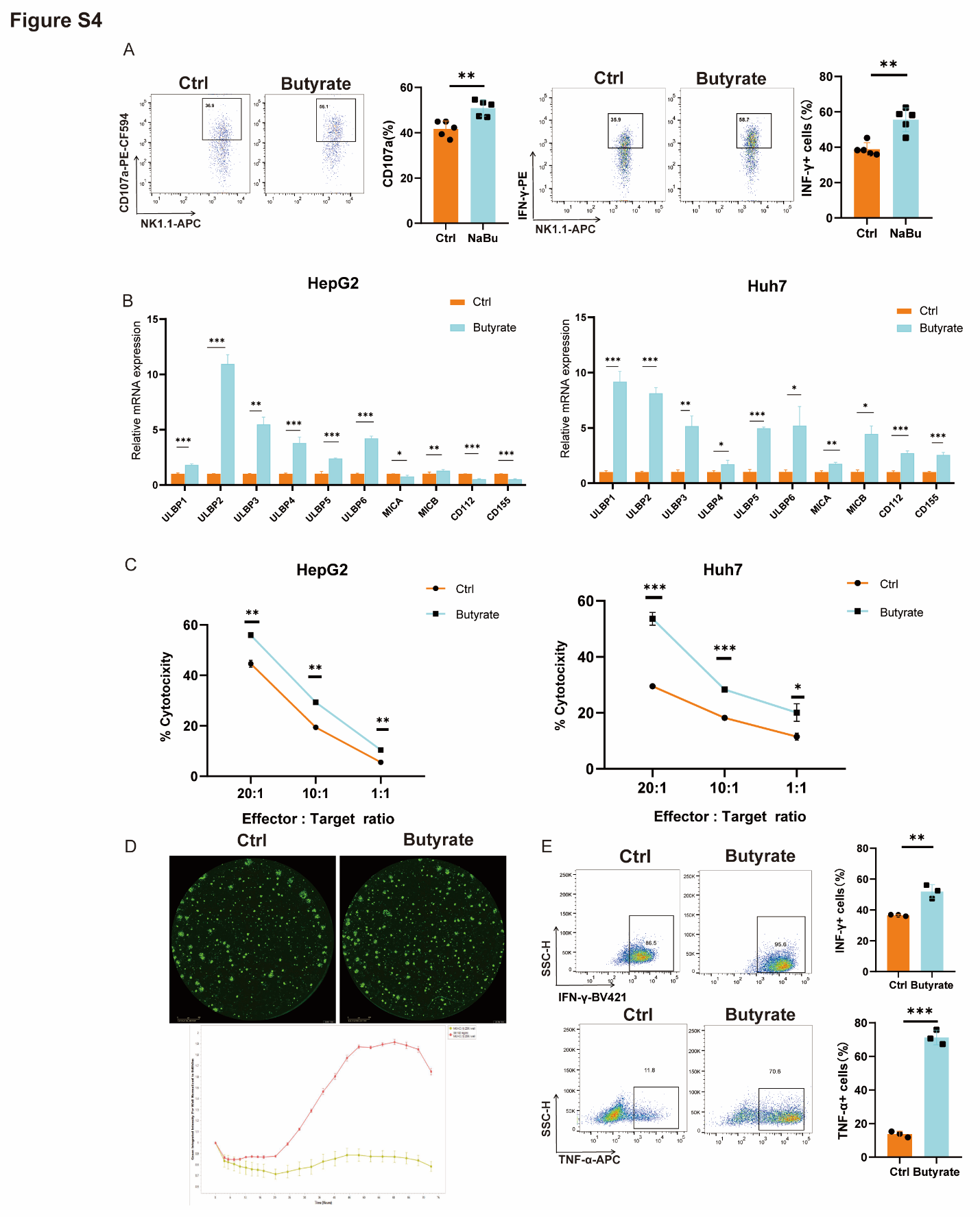


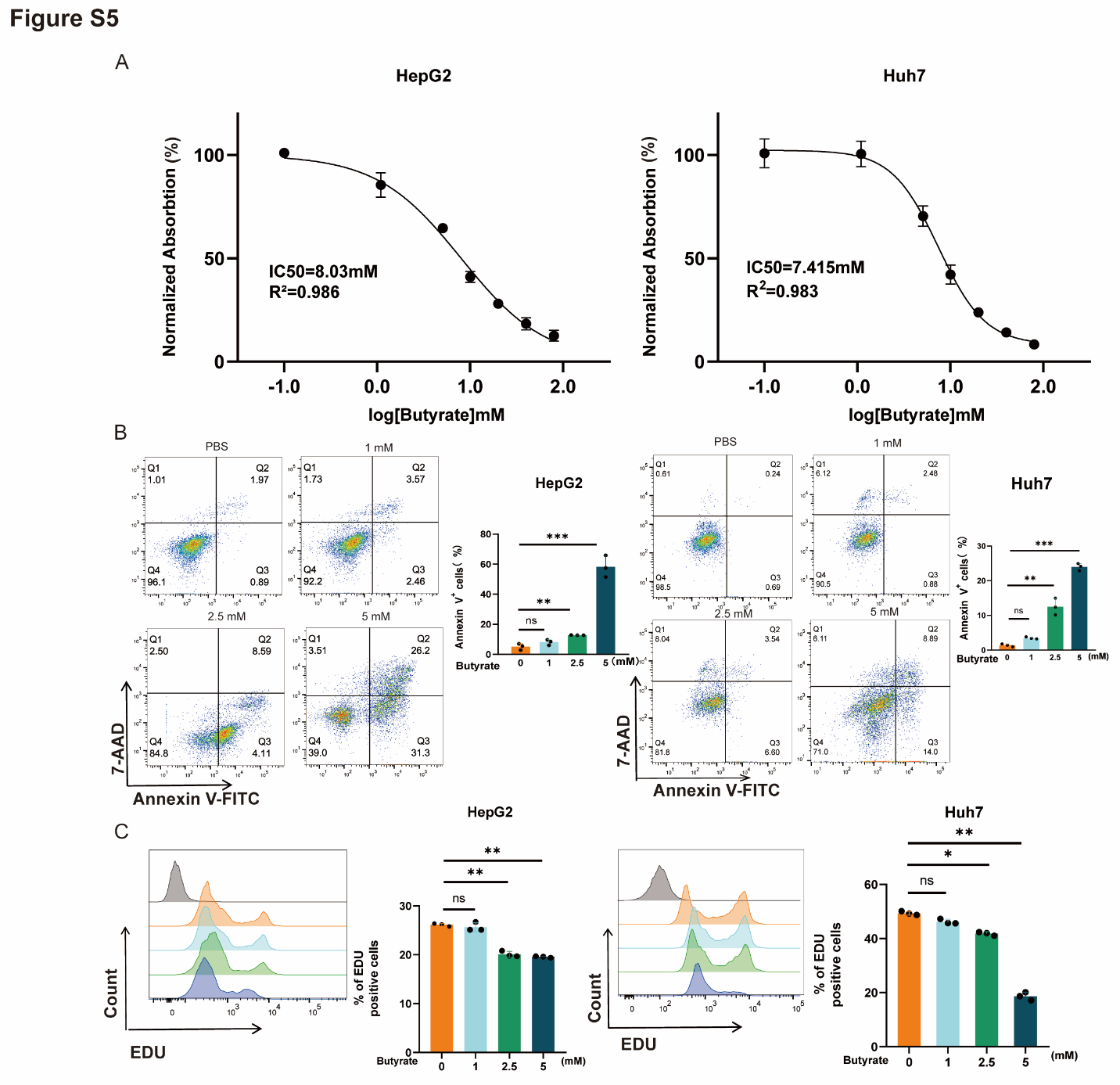


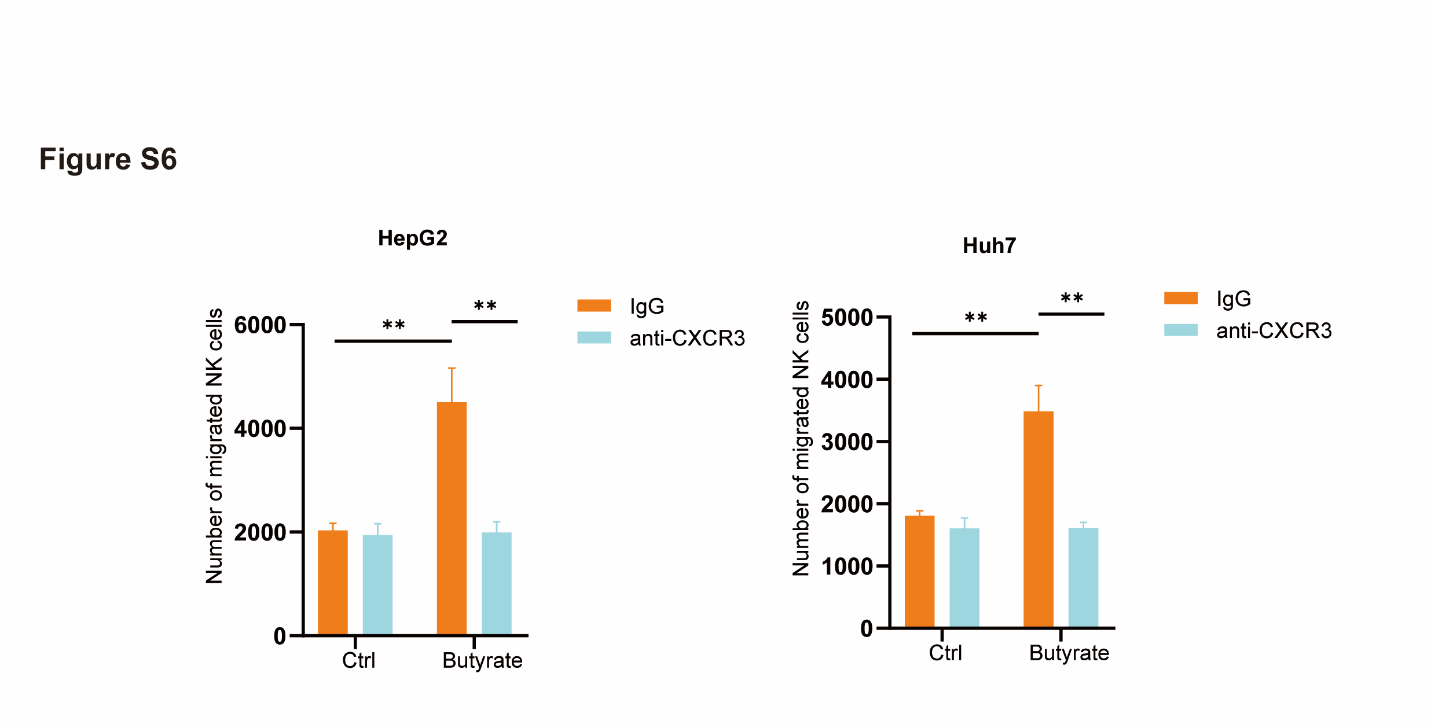


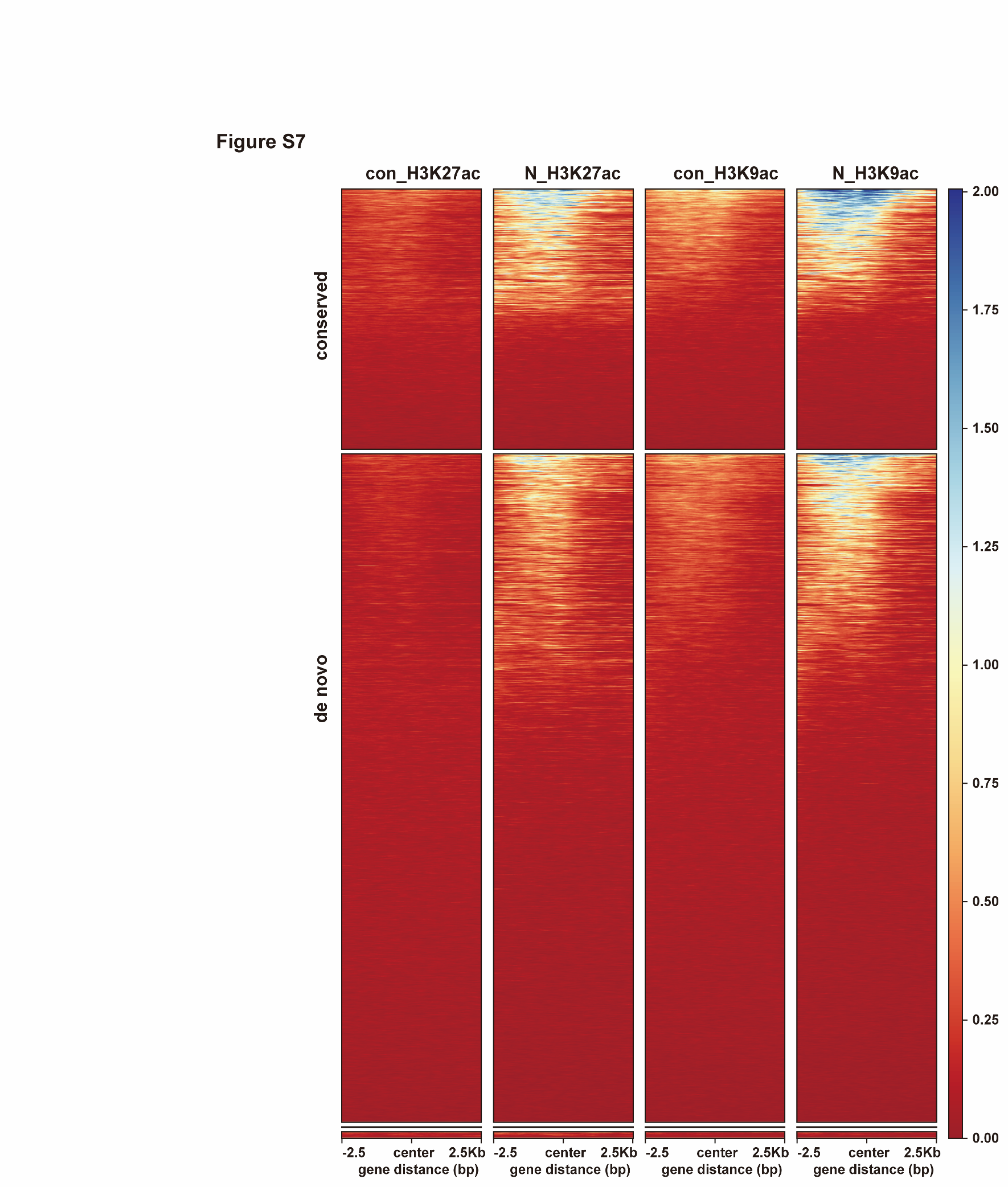

Supplement: Supplemental Material [file KGMI_A_2519706_SM3480.zip › Supp figures.docx]
